# Parental norms and perceptions regarding use of mobile devices by children under five years *

**DOI:** 10.1590/1518-8345.7137.4362

**Published:** 2024-10-25

**Authors:** David San-Martín-Roldán, Adrián González-Marrón, Sonia de-Paz-Cantos, Cristina Lidón-Moyano, Ana Díez-Izquierdo, Jose M Martínez-Sánchez

**Affiliations:** ^1^ Universitat Internacional de Catalunya, Department of Basic Sciences, Barcelona, Catalonia, Spain.; ^2^ Universidad Diego Portales, Facultad de Salud y Odontología, Santiago, Región Metropolitana, Chile.; ^3^ Kenko Lab, Centro de estudios del uso saludable de pantallas, Barcelona, Catalonia, Spain.; ^4^ Instituto de Efectividad Clínica y Sanitaria, Departamento de Evaluación de Tecnologías Sanitarias (ETS) y Economía de la Salud, Buenos Aires, Argentina.; ^5^ Hospital Universitario Vall d’Hebron, Sección de Alergia Pediátrica, Neumología Pediátrica y Fibrosis Quística Infantil, Barcelona, Catalonia, Spain.; ^6^ Universidad de Extremadura, Centro Universitario de Mérida, Group of Evaluation of Health Determinants and Health Policies, Mérida, Badajoz, Spain.

**Keywords:** Mobile Device, Tablet, Smartphone, Preschool Children, Screen Time, Perception

## Abstract

to describe the norms and perceptions of a sample of Spanish parents and guardians on the use of mobile devices (smartphone and/or tablet) by their children and their association with such use.

a quantitative, analytical, cross-sectional, online, exploratory study was conducted with a non-probability sample of parents or guardians of children using smartphones or tablets. The virtual questionnaire consisted of 44 questions and was available at https://www.kenkolab.org/participa/ .

the total number of participants (parents or guardians) was 183 people. It was observed that 26.2% of parents admitted that their children had free and unrestricted access to smartphones or tablets. Associations were identified between time spent using mobile devices and parental attitudes, such as permission without time limitation and use as a reward or punishment. Approximately half of the respondents believe that their children use the mobile device longer than recommended.

most parents exercise some control over their children’s use of devices. Acting on parental norms and perceptions is vital to success in addressing children’s time spent on mobile devices. Community nursing could be a vehicle for education in healthy screen consumption habits in early childhood.

## Highlights

**(1)** Most parents are concerned about their children’s time on mobile devices.

**(2)** Unrestricted access is associated with increased use of mobile devices in children.

**(3)** Parents are concerned about early exposure to mobile devices by children.

**(4)** Parental presence influences children’s mobile device usage time.

**(5)** Need for education and collaboration.

## Introduction

 The apps supply for mobile devices (smartphone and/or tablet) has increased with usage, with the percentage of children aged 3 to 4 years using a mobile device at home rising from 28% in 2013 to 63% in 2019, according to the UK Office of Communications ^(^ 2021 ^)^ . In this sense, mobile devices are replacing computers and are now a central part of children’s lives ^(^ 2021 ^-^ 2021 ^)^ . In the United States, 33% of the screen time of children aged between 0 and 8 years is spent in front of a mobile device ^(^ 2023 ^)^ . In Spain, more than 65% of the population aged 10-15 years has its own cell phone, which means early integration into the Spanish children’s daily life ^(^ 2021 ^,^ 2021b ^)^ . 

 The evidence on the risks and benefits of mobile device use in children is conflicting ^(^ 2020 ^)^ . Among the risks, its regular use is associated with detrimental effects on their physical, neurological, psychological, and social development ^(^ 2021 ^,^ 2023 ^)^ . There is ample evidence that increased use of mobile devices in children is associated with shorter sleep duration, delayed bedtime, sleep problems, poor sleep quality ^(^ 2024 ^)^ and poor nutrition ^(^ 2021b ^,^ 2020 ^)^ . In addition, when combined with low physical activity levels during childhood, screen time increases the risk of obesity ^(^ 2023 ^,^ 2011 ^)^ and depression ^(^ 2021 ^-^ 2020 ^)^ . On the other hand, the use of mobile devices could have positive effects if appropriate content is consumed and in the presence of the child’s parents or caregivers ^(^ 2023 ^,^ 2020 ^)^ . This is the case for high-quality educational programs intended to provide learning opportunities, but only for children older than 2 years ^(^ 2023 ^,^ 2017 ^)^ . In addition, watching a moderate amount of high-quality programming, e.g., “Sesame Street,” improves cognitive, social, and school performance outcomes ^(^ 2019 ^)^ . Smartphones could even be an asset for remaining social when outdoors ^(^ 2021 ^)^ . 

 Parents play a key role in their children’s use and time spent on mobile devices (screen time) ^(^ 2023 ^)^ . However, parents are not usually concerned about the amount of time, but rather the content ^(^ 2023 ^)^ . Also, there is evidence indicating that parents point to several benefits of their children’s use of mobile devices, including their role as an educational and entertainment tool, a device to cope with bedtime, and to promote family bonding and physical activity through active play ^(^ 2023 ^)^ . Moreover, it appears that there are differences in the norms and perceptions, concerns, and approaches used by parents to manage their children’s screen time. Parents who spend more time using screen devices perceive their children’s screen time with less concern ^(^ 2019 ^)^ . Parents who have a positive view of digital technology not only download applications for their children more frequently than other parents, but also try to purchase applications instead of using only free applications. In addition, these parents try to equip their children with at least one type of smart mobile device ^(^ 2021 ^)^ . 

 The inadequate use of mobile devices is an important public health problem in the pediatric population. The health professions’ role, particularly community nursing, with educational interventions has been shown to be effective in reducing screen time in children ^(^ 2023 ^)^ . Given the key role played by parents, the aim of this study was to describe the norms and perceptions of a sample of Spanish parents and guardians about their children’s use of mobile devices (smartphone and/or tablet) and their association with such use. 

## Method

### Study design

Observational, analytical, and cross-sectional study reported following the guidelines of the Strengthening the Reporting of Observational Studies in Epidemiology (STROBE).

### Locus

 Pediatrics Unit of the *Hospital Universitari General de Catalunya* (Sant Cugat del Vallés, Barcelona, Spain) and through dissemination on Instagram (@dospediatrasencasa). 

### Period

The fieldwork was conducted between March 2021 and March 2022.

### Participants

Parents and guardians of children who used mobile devices living in Spain.

### Selection criteria

Parents and guardians of children aged 3 months to 5 years.

### Definition of the sample

Convenience and snowball sample.

### Data collection instruments and study variables

 Data were obtained from a QR (quick response) code that gave access to an online questionnaire. The code was contained in an information leaflet about the study and was given to participants at the pediatrics unit of the *Hospital Universitari General de Catalunya* (Sant Cugat del Vallés, Barcelona, Spain) and via broadcast on Instagram (@dospediatrasencasa). In addition, participants were asked to disseminate the link to the questionnaire among parents of children aged 3 months to 5 years. 

Outcomes were parental norms about children’s use of mobile devices, and perceptions about frequency of use and children’s behavior during or after mobile device use.

 An *ad-hoc* questionnaire was used. The questionnaire consisted of 10 questions in the first part and 13 questions in the second, in addition to 21 questions to address bio-sociodemographic aspects of both the child and the participant. The questionnaire was available online and could be self-completed. 

One set of outcomes were parental norms about the child’s use of mobile devices, perceptions about frequency of use, and child behavior during or after mobile device (smartphone and/or tablet) use. These were assessed through the statements: 1) Your child can use the smartphone or tablet without time limitation; 2) Your child can use the smartphone or tablet without time limitation on the weekends; 3) Your child can use the smartphone or tablet only on weekends; 4) Your child has a time slot per day for smartphone or tablet use; 5) Your child find it more fun to use the smartphone or tablet than to do other activities, such as sports, reading, playing at recess, or other activities; 6) Your child uses the smartphone or tablet longer than he or she should; 7) Your child remains quiet when he or she stops using the smartphone or tablet; 8) Your child cries or screams when he/she must stop using the mobile device. The grade of agreement with the above statements was assessed through Likert scales from strongly agree to strongly disagree. We also assessed the use of mobile devices to keep children entertained in different settings/situations, the use of mobile devices as reward or punishment, and the presence of parents while the child uses the mobile device, through the statements: 1) You use the smartphone or tablet to entertain your son or daughter in situations such as restaurants, doctor’s appointments, public transportation or other; 2) You use the smartphone or tablet as a reward or punishment depending on your child’s behavior; 3) You are present while your son or daughter is using the smartphone or tablet. The grade of agreement with the above statements was also assessed through Likert scales from strongly agree to strongly disagree. Childrens’ daily use of smartphone and tablet was assessed through the questions “Approximately, how much time does your child spend using the following mobile devices each day (Monday through Friday)?” and “Approximately, how much time does your child spend using the following mobile devices on the weekends (Saturday and Sunday)?”. Options to respond both questions included 30-minute categories (“0 minutes”, “30 minutes”, “1 hour”, “1 hour and 30 minutes”, “2 hours”, “2 hours and 30 minutes”, “3 hours”, “3 hours and 30 minutes” and “4 hours or more”). The time of daily use of each device was estimated by weighting the responses to each of the two questions by multiplying them by 5 (weekday school days) and 2 (weekend days), respectively, adding them together and then dividing by 7. For this calculation, the category 4 or more hours was considered as 4.

Sociodemographic variables associated with the child, including gender (male/female), age (less than or equal to 2 years old/from 3 to 5 years old), having at least one sibling (yes/no) and having at least one older sibling (yes/no); and with the parents or guardians, including relationship with the child (mother/father/other), age of the respondent (less than or equal to 35 years old/older than 35 years old) and education level (lower, primary and secondary/university) were also retrieved.

### Data collection

 Baseline data were used from the Smart Screen Health project, which aims to promote healthy use of screen devices in early childhood. The virtual questionnaire consisted of 44 questions and was available at https://www.kenkolab.org/participa/ between March 2021 and March 2022. 

### Data treatment and analysis

Counts and proportions of respondents in agreement (strongly agree + agree) with the statements were estimated overall and according to independent variables. Chi-squared and Fisher’s exact tests were used to explore the association between the grade of agreement and independent variables. The normality of the variable time of mobile device use was assessed using Kolmogorov-Smirnov and Shapiro-Wilk tests, as well as Q-Q plots. Due to non-normality of data, time of mobile device use was described with median (Me) and interquartile range. Mann-Whitney tests or Kruskal-Wallis tests were used to estimate significant differences in distributions of mobile device use time between respondents who agreed (strongly agree + agree) with the statements and those who did not, overall (i.e., children who used smartphone or tablet) and for smartphone and tablet exclusive users. To perform the statistical analyses, the statistical program R version 4.0.2 was used. Two-tailed tests were carried out and a significance level of 0.05 was set.

### Ethical aspects

 The study was evaluated and approved by the Clinical Research Ethics Committee of *Grupo Hospitalario Quirónsalud-Catalunya* , on 10/02/2021 (minute nº03/2021). Written informed consent was requested after reading the information sheet and before giving access to the survey. 

## Results

The final sample consisted of a total of 183 respondents, who were parents or guardians of children using either smartphones or tablets, or both. An estimated 54.1% of the respondents were fathers, mothers, or guardians of male children; 89.1% were mothers and 78.1% had university studies.

 In relation to the usage rules and restrictions, an estimated 1.6% (n=3) and 3.8% (n=7) of respondents agreed that the child could use the smartphone or tablet with no time limitation and no time limitation on weekends, respectively ( Table 1 ). The median use of both mobile devices (smartphone and tablet) by children was significantly higher in those who adopt a position in favor of their child being able to use them without time limitation (Me=103.0 minutes/day), compared to children whose parents do not adopt such a position. Specifically, this time is significantly higher when the use is only on smartphones (Me=77.1 minutes/day). In contrast, it presents lower minutes in those who stipulate that their child can only use the smartphone or tablet on weekends (Me=23.6 minutes/day) ( Table 2 ). 

 Also, 18.6% (n=34) of respondents agreed that the child could use the smartphone or tablet only on weekends and 24.0% (n=44) that the child had one time slot per day to use the device ( Table 1 ), whereas 91.8% (n=168) agreed that the parent was present while using the screen device ( Table 3 ). The overall median usage times were significantly higher for children who used the mobile device without the father present compared to those who used it with the father present (p-value = 0.042) ( Table 4 ). In terms of perceptions and frequency of use, 48.1% (n=88) of respondents agreed that the child used the mobile device longer than he/she should and 17.5% (n=32) agreed that the child found it more fun to use the smartphone or tablet than to do other activities (e.g., playing sports, reading, playing at recess) ( Table 1 ). Some 17.5% (n=32) and 2.7% (n=5) of respondents used the mobile device to keep the child entertained in different environments and as a reward or punishment. ( Table 3 ). Overall median times of use were significantly higher in children of parents who used these strategies as compared with those who did not (p-value <0.001 and p-value 0.008, respectively) ( Table 4 ). 

## Discussion

 According to our results, parents, and guardians of the children in our sample appear to be concerned, in general, about the use and screen time spent by children on mobile devices (smartphone and/or tablet). Screen time during early childhood has been a pediatric and public health concern ^(^ 2023 ^)^ . This may be one of the factors contributing to disparities in early childhood development. 

 The parental norm of children’s unrestricted access to mobile devices is associated with increased daily usage time, being 2.6 times higher compared to those without free access, which may have consequences for children’s cognitive, social, and emotional development ^(^ 2023 ^)^ . The fact that children under 2 years of age use mobile devices more than 30 minutes a day, and that this time increases considerably when they have free access, does not meet any screen time recommendations, and raises concerns about the impact of early exposure to technology on child development ^(^ 2023 ^)^ . A very low proportion of parents use the smartphone or tablet as a reward or punishment, with median use of 2 hours per day, depending on the child’s behavior. Reassuringly, the majority do not resort to this disciplinary strategy, but those who use it for noncompliance with recommendations are of concern. 

 Parental presence in device use was associated with having a sibling, but not with having an older sibling. Another study indicated that the presence of a sibling and less personal living space were found to be significant predictors of children’s screen time ^(^ 2021 ^)^ . Parental control-oriented management of smartphone use is not very effective and aggravates smartphone addiction considering that parents who spend more time in front of a screen perceive their children’s screen time with less concern ^(^ 2019 ^,^ 2021 ^)^ . This suggests that home and community sociocultural and environmental factors, in addition to parental behavioral factors, such as their knowledge about the health effects of mobile devices, may favor children’s use. Healthcare institutions and healthcare personnel, especially nurses, should take up the challenge of raising awareness among children and adolescents about the responsible use of mobile devices ^(^ 2021 ^)^ . Even more so if we consider that as 

**Table 1:** - Overall and stratified counts and percentages of parents’ and guardians’ agreement with mobile device (smartphone/tablet) norms of use, and perceptions of frequency of use and child behavior during or after use (n = 183). Spain, 2021-2022

	**n**	Q1* n (%)	**p** ^†^	**(95% CI)** ^‡^	**Q2** ^§^ **n (%)**	**p** ^†^	**(95% CI)** ^‡^	**Q3** ^||^ **n (%)**	**p** ^†^	**(95% CI)** ^‡^	**Q4** ^¶^ **n (%)**	**p** ^†^	**(95% CI)** ^‡^
Total	183	3 (1.6)			7 (3.8)			34 (18.6)			44 (24.0)		
Child’s gender			0.305	(-0.08 to 0.01)		0.581	(-0.09 to 0.04)		0.421	(-0.08 to 0.01)		0.103	(-0.24 to 0.01)
Male	99	3 (3.0)			5 (5.1)			21 (21.2)			29 (29.3)		
Female	84	0 (0.0)			2 (2.4)			13 (15.5)			15 (17.9)		
Child’s age			1.000	(-0.06 to 0.04)		0.801	(-0.04 to 0.09)		0.006	(0.05 to 0.28)		0.874	(-0.15 to 0.11)
Less than or equal to 2 years old	100	2 (2.0)			3 (3.0)			11 (11.0)			25 (25.0)		
From 3 to 5 years old	83	1 (1.2)			4 (4.8)			23 (27.7)			19 (22.9)		
Siblings			0.946	(-0.06 to 0.03)		0.099	(-0.13 to -0.0018		0.050	(-0.24 to -0.01)		0.819	(-0.10 to 0.15)
Yes	88	2 (2.3)			6 (6.9)			22 (25.0)			20 (22.7)		
No	95	1 (1.1)			1 (1.1)			12 (12.6)			24 (25.3)		
Older siblings			0.567	(-0.09 to 0.02)		0.089	(-0.15 to 0.00)		0.055	(-0.26 -0.01)		0.813	(-0.11 to 0.15)
Yes	63	2 (3.2)			5 (7.9)			17 (26.9)			14 (22.2)		
No	120	1 (0.8)			2 (1.7)			17 (14.2)			30 (25.0)		
Relationship with the child			1.000	(-0.05 to 0.11)		0.831	(-0.08 to 0.09)		0.727	(-0.14 to 0.24)		1.000	(-0.16 to 0.24)
Mother	163	3 (1.8)			7 (4.3)			30 (18.4)			39 (23.9)		
Father	17	0 (0.0)	-		0 (0.0)			2 (11.8)	-		4 (23.5)	-	
Other	3	0 (0.0)			0 (0.0)			2 (66.7)			1 (33.3)		
Respondent’s age			0.225	(-0.09 to 0.01)		0.398	(-0.10 to 0.03)		0.124	(-0.01 to 0.21)		0.315	(-0.05 to 0.20)
Less than or equal to 35 years old	89	3 (3.4)			5 (5.6)			12 (13.5)			18 (20.2)		
Older than 35 years old	94	0 (0.0)			2 (2.1)			22 (23.4)			26 (27.7)		
Level of education			0.234	(-0.15 to 0.09)		1.000	(-0.12 to 0.05)		0.158	-0.28 to 0.03)		0.639	(-0.11 to 0.18)
Lower, primary or secondary	40	2 (5.0)			2 (5.0)			11 (27.5)			8 (20.0)		
University studies	143	1 (0.7)			5 (3.5)			23 (16.1)			36 (25.2)		
	**n**	Q5** n (%)	**p** ^†^	**(95% CI)** ^‡^	**Q6** ^††^ **n (%)**	**p** ^†^	**(95% CI)** ^‡^	**Q7** ^‡‡^ **n (%)**	**p** ^†^	**(95% CI)** ^‡^	**Q8** ^§§^ **n (%)**	**p** ^†^	**(95% CI)** ^‡^
Total	183	32 (17.5)			88 (48.1)			100 (54.6)			54 (29.5)		
Child’s gender			0.941	(-0.13 to 0.10)		0.080	(-0.28 to 0.01)		1.000	(-0.14 to 0.15)		0.456	(-0.19 to 0.07)
Male	99	18 (18.2)			54 (54.6)			54 (54.6)			32 (32.3)		
Female	84	14 (16.7)			34 (40.5)			46 (54.8)			22 (26.2)		
Child’s age			0.431	(-0.17 to 0.06)		0.286	(-0.06 to 0.23)		0.124	(-0.02 to 0.27)		0.051	(-0.27 to -0.01)
Less than or equal to 2 years old	100	20 (20.0)			44 (44.0)			49 (49.00)			36 (36.0)		
From 3 to 5 years old	83	12 (14.5)			44 (53.0)			51 (61.5)			18 (21.7)		
Siblings			0.410	(-0.17 to 0.05)		0.067	(-0.29 to -0.00)		0.056	(-0.29 to -0.01)		0.862	(-0.16 to 0.11)
Yes	88	18 (20.5)			49 (55.7)			55 (62.5)			27 (30.7)		
No	95	14 (14.7)			39 (41.1)			45 (47.4)			27 (28.4)		
Older siblings			0.543	(-0.18 to 0.07)		0.053	(-0.31 -0.01)		0.202	(-0.26 to 0.04)		1.000	(-0.15 to 0.13)
Yes	63	13 (20.6)			37 (58.7)			39 (61.9)			19 (30.2)		
No	120	19 (15.8)			51 (42.5)			61 (50.8)			35 (29.2)		
Relationship with the child			0.288	(-0.04 to 0.37)		0.381	(-0.35 to 0.09)		0.014	(-0.46 to -0.04)		0.823	(-0.18 to 0.24)
Mother	163	26 (15.9)			81 (49.7)			94 (57.7)			48 (29.5)		
Father	17	5 (29.4)	-		6 (35.3)	-		4 (23.5)	-		6 (35.3)	-	
Other	3	1 (33.3)			1 (33.3)			2 (66.7)			0 (0.0)		
Respondent’s age			0.678	(-0.08 to 0.15)		1.000	(-0.15 to 0.14)		0.579	(-0.20 to 0.09)		0.567	(-0.08 to 0.18)
Less than or equal to 35 years old	89	14 (15.7)			43 (48.3)			51 (57.3)			24 (26.9)		
Older than 35 years old	94	18 (19.2)			45 (47.9)			49 (52.1)			30 (31.9)		
Level of education			0.811	(-0.19 to 0.09)		0.924	(-0.20 to 0.15)		0.625	(-0.11 to 0.23)		0.366	(-0.07 to 0.23)
Lower, primary or secondary	40	8 (20.0)			20 (50.0)			20 (50.0)			9 (22.5)		
University studies	143	24 (17.8)			68 (47.6)			80 (55.9)			45 (31.5)		

* Q1 = Your child can use the smartphone or tablet without time limitation

^†^ p = p-value (each p-value comes from the chi-square value by Fisher’s test)

^‡^ 95%CI = 95% Confidence interval

^§^ Q2 = Your child can use the smartphone or tablet without time limitation on the weekends

^||^ Q3 = Your child can use the smartphone or tablet only on weekends

^¶^ Q4 = Your child has a time slot per day for smartphone or tablet use

** Q5 = Your child find it more fun to use the smartphone or tablet than to do other activities, such as sports, reading, playing at recess, or other activities

^††^ Q6 = Your child uses the smartphone or tablet longer than he or she should

^‡‡^ Q7 = Your child remains quiet when he or she stops using the smartphone or tablet

^§§^ Q8 = Your child cries or screams when he/she must stop using the smartphone or tablet

**Table 2:** - Overall and device-stratified medians and quartiles of time of use (in minutes per day) in children according to agreement with mobile device use norms and with perceptions of frequency of use and of child behavior while or after use, according to parents and guardians (n = 183). Spain, 2021-2022

	**Smartphone and/or tablet (n=183)**	**Only smartphone (n=139)**	**Only tablet (n=80)**
	Median (IQR*)	Median (IQR*)	Median (IQR*)
Q1 ^†^			
Agree ^‡^	103.0 (90.0-111.0)	77.1 (68.6-90.0)	60.0 (60.0-60.0)
Rest ^§^	30.0 (17.1-51.4)	30.0 (8.57-30.0)	30.0 (17.1-60.0)
p ^||^	0.010	0.005	0.912
Q2 ^¶^			
Agree ^‡^	55.7 (27.9-90.0)	60.0 (30.0-77.1)	17.1 (8.57-47.1)
Rest ^§^	30.0 (17.1-51.4)	30.0 (8.57-30.0)	30.0 (17.1-62.1)
p ^||^	0.164	0.402	0.305
Q3**			
Agree ^‡^	23.6 (8.6-54.6)	17.1 (8.57-34.3)	21.4 (8.6-55.7)
Rest ^§^	30.0 (21.4-55.7)	30.0 (17.1-38.6)	30.0 (24.6-65.4)
p ^||^	0.102	0.026	0.167
Q4 ^††^			
Agree ^‡^	30.0 (8.6-55.7)	27.9 (8.6-30.0)	30.0 (23.6-60.0)
Rest ^§^	30.0 (19.3-53.6)	30.0 (17.1-38.6)	30.0 (17.1-60.0)
p ^||^	0.527	0.012	0.215
Q5 ^‡‡^			
Agree ^‡^	34.3 (17.1-63.2)	30.0 (8.57-38.6)	34.3 (25.7-58.9)
Rest ^§^	30.0 (17.1-40.7)	30.0 (8.57-30.0)	30.0 (10.7-60.0)
p ^||^	0.256	0.773	0.101
Q6 ^§§^			
Agree ^‡^	38.6 (30.0-66.4)	30.0 (21.43-60.0)	34.3 (17.1-73.9)
Rest ^§^	30.0 (8.6-30.0)	30.0 (8.57-30.0)	30.0 (8.57-40.7)
p ^||^	< 0.001	0.005	0.038
Q7 ^||||^			
Agree ^‡^	30.0 (20.4-60.0)	30.0 (8.57-38.6)	30.0 (22.5-71.8)
Rest ^§^	30.0 (17.1-38.6)	30.0 (8.57-30.0)	30.0 (8.57-50.4)
p ^||^	0.047	0.627	0.024
Q8 ^¶¶^			
Agree ^‡^	30.0 (17.1-48.2)	30.0 (17.1-38.6)	30.0 (8.57-60.0)
Rest ^§^	30.0 (17.1-55.7)	30.0 (8.57-30.0)	30.0 (24.6-61.1)
p ^||^	0.970	0.702	0.277

* IQR = Interquartile range

^†^ Q1 = Your child can use the smartphone or tablet without time limitation

^‡^ Agree = Totally agree + Agree

^§^ Rest = Neither agree nor disagree + Disagree + Strongly Disagree

^||^ p = p-value (each p-value is from the Mann-Whitney U test)

^¶^ Q2 = Your child can use the smartphone or tablet without time limitation on the weekends

** Q3 = Your child can use the smartphone or tablet only on weekends

^††^ Q4 = Your child has a time slot per day for smartphone or tablet use

^‡‡^ Q5 = Your child find it more fun to use the smartphone or tablet than to do other activities, such as sports, reading, playing at recess, or other activities

^§§^ Q6 = Your child uses the smartphone or tablet longer than he or she should

^||||^ Q7 = Your child remains quiet when he or she stops using the smartphone or tablet

^¶¶^ Q8 = Your child cries or screams when he/she must stop using the smartphone or tablet

 children grow older, the level of autonomy and independence in the use of mobile devices also increases, which coincides with this study’s results ^(^ 2017 ^,^ 2024 ^)^ . 

 Older parents, who report more mobile device use time by their children, may face additional challenges in terms of setting limits and promoting balance between screen time and other activities, as it was evidenced that the older the age of parents and the more time parents spend viewing mobile devices is associated with more screen time in children ^(^ 2023 ^)^ . Differences in time spent using 

**Table 3:** - Counts and percentages of agreement with strategies of using mobile devices (smartphone/tablet) to keep children entertained in different environments, use of mobile devices as reward or punishment, in parents and guardians (n = 183). Spain, 2021-2022

	**n**	Use of the mobile device as entertainment in different settings* n (%)	**p** ^†^	**(95% CI)** ^‡^	**Use of mobile device as reward/punishment n (%)**	**p** ^†^	**(95% CI)** ^‡^	**Parent presence while using mobile device n (%)**	**p** ^†^	**(95% CI)** ^‡^
Total	183	32 (17.5)			5 (2.7)			168 (91.8)		
Child’s gender			0.213	(-0.19 to 0.03)		1.000	(-0.06 to 0.05)		0.453	(-0.04 to 0.12)
Male	99	21 (21.2)			3 (3.0)			89 (89.0)		
Female	84	11 (13.1)			2 (2.4)			79 (94.0)		
Child’s age			0.437	(-0.06 to 0.17)		0.832	(-0.04 to 0.08)		0.144	(-0.16 to 0.01)
Less than or equal to 2 years old	100	15 (15.0)			2 (2.0)			95 (95.0)		
From 3 to 5 years old	83	17 (20.5)			3 (3.6)			73 (88.0)		
Siblings			0.410	(-0.17 to 0.05)		0.320	(-0.10 to 0.02)		0.020	(0.03 to 0.19)
Yes	88	18 (20.5)			4 (4.5)			76 (86.4)		
No	95	14 (14.7)			1 (1.1)			92 (96.8)		
Older siblings			0.153	(-0.23 to 0.02)		0.089	(-0.14 to -0.00)		1.000	(-0.09 to 0.09)
Yes	63	15 (23.8)			4 (6.3)			58 (92.1)		
No	120	17 (14.2)			1 (0.8)			110 (91.7)		
Relationship to child			0.750	(-0.12 to 0.25)		1.000	(-0.07 to 0.10)		0.004	(-0.41 to -0.03)
Mother	163	28 (17.2)			5 (3.1)			153 (93.9)		
Father	17	4 (23.5)			0 (0.0)			12 (70.6)		
Other	3	0 (0.0)			0 (0.0)			3 (100.0)		
Respondent’s age			0.006	(-0.27 to -0.05)		0.060	(-0.12 to -0.01)		1.000	(-0.09 to 0.08)
Less than or equal to 35 years old	89	23 (25.8)			5 (5.6)			82 (92.1)		
Older than 35 years old	94	9 (9.6)			0 (0.0)			86 (91.5)		
Level of education			0.238	(-0.26 to 0.04)		1.000	(-0.09 to 0.05)		0.885	(-0.07 to 0.15)
Lower, primary and secondary	40	10 (25.0)			1 (2.5)			36 (90.0)		
University	143	22 (15.4)			4 (2.8)			132 (92.3)		

* Different Settings = Restaurants, doctor’s appointment, public transportation or other

^†^ p = p-value (each p-value comes from the chi-square value by Fisher’s test)

^‡^ 95%CI = 95% Confidence interval

**Table 4:** - Medians and quartiles of time (in minutes per day) of use according to mobile device in children and daily strategies of mobile device use, use as reward/punishment and parental presence, in parents and guardians (n = 183). Spain, 2021-2022

	**Smartphone & tablet**	**Only smartphone**	**Only tablet**
	Median (IQR*)	Median (IQR*)	Median (IQR*)
Use of the mobile device as entertainment in different situations ^†^			
Agree ^‡^	49.3 (30.0-80.4)	30.0 (17.1-47.1)	53.6 (28.9-90.0)
Rest ^§^	30.0 (17.1-38.6)	30.0 (8.57-30.0)	30.0 (10.7-55.7)
p ^||^	< 0.001	< 0.001	0.510
Use of mobile device as reward/punishment			
Agree ^‡^	120.0 (55.7-163.0)	30.0 (30.0-60.0)	81.4 (51.4-114.0)
Rest ^§^	30.0 (17.1-50.4)	30 (8.57-36.4)	30.0 (15.0-60.0)
p ^||^	0.008	0.051	0.020
Parent presence while using mobile device			
Agree ^‡^	30.0 (17.1-48.2)	30.0 (8.57-30.0)	30.0 (8.57-60.0)
Rest ^§^	47.1 (23.6-101.0)	30.0 (8.57-60.0)	38.6 (25.7-75.0)
p ^||^	0.042	0.668	0.005

* IQR = Interquartile range

^†^ Different Situations = Restaurants, doctor’s appointment, public transportation or other

^‡^ Agree = Totally agree +Agree

^§^ Rest = Neither agree nor disagree + Disagree + Strongly Disagree

^||^ p = p-value (each p-value is from the Mann-Whitney U test)

mobile devices may reflect different attitudes and perceptions towards technology in older parents, who may have a more permissive view or value the potential benefits of technology more highly. On the other hand, younger parents may be more cautious or restrictive in terms of time spent using mobile devices. Favorably, most parents (91.8%) are present while their children are using mobile devices, indicating active supervision and parental permissiveness during mobile device use. We found associations such as the use of mobile devices exclusively on weekends with the age of the child and the relationship with the child (mother or father), explained by the fact that Spanish society has undergone drastic changes in lifestyle in recent decades.

 Another association was the perception that the child remains calm when he or she stops using the mobile device and the relationship with the child (mother or father), which points to the idea that some parents believe that use may have a positive impact on their child’s behavior. The associations show that this new health determinant requires different interventions in view of the risks involved. Almost a quarter of Spanish children aged 1-14 years who exceeded 120 minutes of daily screen time in leisure activities experienced a reduction in sleep duration ^(^ 2021a ^)^ . 

 Thus far, studies have not focused on studying the norms and perceptions of parents and caregivers in Europe regarding the use of screen devices. Almost all are found in Asian and Anglo-Saxon countries ^(^ 2023 ^,^ 2021 ^,^ 2023 ^,^ 2011 ^)^ . To our knowledge, this is one of the first studies describing the norms and perceptions of children’s parents using mobile devices, leaving behind the television paradigm. The inclusion of fathers, mothers, and guardians, as well as the variability in educational levels, provides a broad perspective and allows for more general results. The combination of numerical data and perceptual responses provides a more complete understanding of children’s use of mobile devices. The study addresses the issue of children’s access to and use of mobile devices, which is relevant today and may have implications for parenting and child development. 

 In the future, a dramatic increase in the use of mobile devices by children is expected. This underscores the importance of providing education and support to parents regarding screen use during the early years of life, considering the realities of today’s society ^(^ 2023 ^)^ . There is evidence that nurses can intervene in the framework of parental education for compliance with recommendations on screen time in children, taking into account that this is a social determinant of health in our times ^(^ 2023 ^,^ 2022 ^)^ . Collaboration between researchers, policy makers, and parents is essential to update recommendations for pediatricians and nurses to appropriately manage screen time during early childhood ^(^ 2021 ^,^ 2023 ^,^ 2024 ^)^ . 

 The present study has some limitations. Firstly, the selection of participants could have generated biases and limited the representativeness of the sample. Also, we did not inquire about the use of mobile devices by parents, which could influence their children’s time of use ^(^ 2023 ^,^ 2019 ^)^ . No information is provided on the development and validation process of the questionnaire, which raises doubts about the reliability and validity of the measures used. The assessment of usage time in 30-minute intervals may be imprecise and depend on the participants’ memory. In the last category we have limited it to a maximum of 4 hours. Therefore, if there are children who have used more than that time, we have not taken them into account. This study did not ascertain the qualitative aspects of the phenomenon and was a participant self-report of mobile device use time in children, as it considered the caregiver’s statement, however, this supports the idea of further research in this aspect. 

## Conclusion

Parental rules influence the time children spend on mobile devices. Unlimited permissions result in more screen time, with possible effects on children’s development. Parental supervision also matters; without it, children spend more time in front of screens. Many parents admit that their children exceed recommended limits and prefer devices to other activities. These perceptions influence screen time, highlighting the need for parental supervision and early education. Health professionals, such as nurses, can play a key role in two main areas: educating parents to achieve the recommendations and promoting healthy screen time patterns in children and adolescents.
