# Supplementary material for: Parental norms and perceptions regarding use of mobile devices by children under five years
Source: Rev Lat Am Enfermagem. 2024 Oct 25;32:e4362. doi: 10.1590/1518-8345.7137.4362 (PMC11526287; doi:10.1590/1518-8345.7137.4362)
Supplement: Translation

# Translation: Normas y percepciones de los padres sobre el uso de dispositivos móviles por parte de niños menores de cinco años *

**DOI:** 10.1590/1518-8345.7137.4361

**Published:** 2024-10-09

**Authors:** David San-Martín-Roldán, Adrián González-Marrón, Sonia de-Paz-Cantos, Cristina Lidón-Moyano, Ana Díez-Izquierdo, Jose M Martínez-Sánchez

**Affiliations:** ^1^ Universitat Internacional de Catalunya, Department of Basic Sciences, Barcelona, Catalonia, España.; ^2^ Universidad Diego Portales, Facultad de Salud y Odontología, Santiago, Región Metropolitana, Chile.; ^3^ Kenko Lab, Centro de estudios del uso saludable de pantallas, Barcelona, Catalonia, España.; ^4^ Instituto de Efectividad Clínica y Sanitaria, Departamento de Evaluación de Tecnologías Sanitarias (ETS) y Economía de la Salud, Buenos Aires, Argentina.; ^5^ Hospital Universitario Vall d’Hebron, Sección de Alergia Pediátrica, Neumología Pediátrica y Fibrosis Quística Infantil, Barcelona, Catalonia, España.; ^6^ Universidad de Extremadura, Centro Universitario de Mérida, Group of Evaluation of Health Determinants and Health Policies, Mérida, Badajoz, España.

**Keywords:** Dispositivo Móvil, Tableta, Teléfono Inteligente, Preescolares, Tiempo de Pantalla, Percepción

## Abstract

describir las normas y percepciones de una muestra de padres y tutores españoles sobre el uso de dispositivos móviles ( *smartphones* y/o *tablets* ) por parte de sus hijos y su asociación con este uso.

estudio cuantitativo, analítico, transversal, *online* y exploratorio con una muestra no probabilística de padres o tutores de niños que utilizan *smartphones* o *tablets* . El cuestionario virtual constaba de 44 preguntas y se encontraba disponible en https://www.kenkolab.org/participa/ .

el número total de participantes (padres o tutores) fue de 183 personas. Se observó que el 26,2% de los padres admitieron que sus hijos tenían acceso gratuito y sin restricciones a *smartphones* o *tablets* . Se identificaron asociaciones entre el tiempo dedicado al uso de dispositivos móviles y las actitudes de los padres, como el permiso sin limitaciones de tiempo y el uso como recompensa o castigo. Aproximadamente la mitad de los encuestados considera que sus hijos utilizan su dispositivo móvil por más tiempo que el recomendado.

la mayoría de los padres ejercen cierto control sobre el uso de dispositivos de sus hijos. Actuar según las normas y percepciones de los padres es vital para abordar con éxito el tiempo que los niños pasan en los dispositivos móviles. La enfermería comunitaria puede ser un medio para la educación sobre hábitos saludables de consumo de pantallas en la primera infancia.

## Destacados

**(1)** La mayoría de los padres están preocupados por el tiempo que sus hijos destinan al uso de dispositivos móviles.

**(2)** El acceso irrestricto se asocia con un mayor uso de dispositivos móviles por parte de los niños.

**(3)** Los padres se preocupan por la exposición temprana de los niños a los dispositivos móviles.

**(4)** La presencia de los padres influye en el tiempo que los niños destinan al uso de los dispositivos móviles.

**(5)** Necesidad de educación y colaboración.

## Introducción

 La oferta de aplicaciones para dispositivos móviles ( *smartphones* y/o *tablets* ) ha aumentado con el uso, habiéndose incrementado el porcentaje de niños entre 3 y 4 años que utilizan un dispositivo móvil en casa del 28% en 2013 al 63% en 2019, según la Oficina de Comunicaciones del Reino Unido ( *UK Office of Communications* ) ^(^ 2021 ^)^ . En este sentido, los dispositivos móviles están reemplazando a las computadoras y ahora son parte central de la vida de los niños ^(^ 2021 ^-^ 2021 ^)^ . En el caso de los niños de 0 a 8 años, el 33% del tiempo ante una pantalla lo pasan frente a un dispositivo móvil en los Estados Unidos ^(^ 2023 ^)^ . En España, más del 65% de la población de entre 10 y 15 años dispone de teléfono móvil propio, lo que implica una integración temprana en la vida cotidiana de los niños españoles ^(^ 2021 ^,^ 2021b ^)^ . 

 La evidencia sobre los riesgos y beneficios del uso de dispositivos móviles en niños es contradictoria ^(^ 2020 ^)^ . Entre los riesgos, su uso regular está asociado a efectos nocivos sobre el desarrollo físico, neurológico, psicológico y social ^(^ 2021 ^,^ 2023 ^)^ . Se ha demostrado que el mayor uso de dispositivos móviles en los niños se asocia con una duración reducida del sueño, retrasos en la hora de acostarse, problemas de sueño, mala calidad del sueño ^(^ 2024 ^)^ y mala nutrición ^(^ 2021b ^,^ 2020 ^)^ . Además, cuando se combina con bajos niveles de actividad física durante la infancia, el tiempo frente a una pantalla aumenta el riesgo de obesidad ^(^ 2023 ^,^ 2011 ^)^ y depresión ^(^ 2021 ^-^ 2020 ^)^ . Por otro lado, el uso de dispositivos móviles puede tener efectos positivos si se absorbe el contenido adecuado en presencia de los padres o cuidadores del niño ^(^ 2023 ^,^ 2020 ^)^ . Este es el caso de los programas educativos de alta calidad diseñados para brindar oportunidades de aprendizaje, pero solo para niños mayores de 2 años ^(^ 2023 ^,^ 2017 ^)^ . Además, ver una cantidad moderada de programas de alta calidad, por ejemplo, “ *Vila Sésamo* ”, mejora los resultados cognitivos, sociales y de rendimiento académico ^(^ 2019 ^)^ . Los *smartphones* pueden incluso ser un recurso para socializar cuando se está al aire libre ^(^ 2021 ^)^ . 

 Los padres juegan un papel fundamental en el uso que hacen sus hijos de los dispositivos móviles y el tiempo que pasan frente a ellos (tiempo de pantalla) ^(^ 2023 ^)^ . Sin embargo, a los padres generalmente no les preocupa la cantidad de tiempo, sino el contenido ^(^ 2023 ^)^ . Además, hay evidencia que indica que los padres señalan varios beneficios del uso de dispositivos móviles por parte de sus hijos, incluida su función como herramienta educativa y de entretenimiento, como dispositivo para gestionar la hora de acostarse y para promover los vínculos familiares y la actividad física a través del juegos activos ^(^ 2023 ^)^ . Además, parece haber diferencias en las normas, percepciones, preocupaciones y enfoques utilizados por los padres para gestionar el tiempo que sus hijos pasan frente a la pantalla. Los padres que pasan más tiempo usando dispositivos con pantalla perciben el tiempo que sus hijos pasan frente a la pantalla con menor preocupación ^(^ 2019 ^)^ . Los padres que tienen una visión positiva de la tecnología digital no sólo descargan aplicaciones para sus hijos con más frecuencia que otros padres, sino que también intentan comprar aplicaciones en lugar de utilizar apenas las aplicaciones gratuitas. Además, estos padres intentan equipar a sus hijos con al menos un tipo de dispositivo móvil inteligente ^(^ 2021 ^)^ . 

 El uso inadecuado de dispositivos móviles es un importante problema de salud pública en la población pediátrica. Se ha demostrado que el papel de las profesiones de la salud, en especial de la enfermería comunitaria, con intervenciones educativas, es eficaz para reducir el tiempo frente a la pantalla en los niños ^(^ 2023 ^)^ . Dado el papel fundamental que desempeñan los padres, el objetivo de este estudio fue describir las normas y percepciones de una muestra de padres y tutores españoles sobre el uso de dispositivos móviles ( *smartphones* y/o *tablets* ) por parte de sus hijos y su asociación con este uso. 

## Método

### Diseño del estudio

 Estudio observacional, analítico y transversal de conformidad con los lineamientos *del Strengthening the Reporting of Observational Studies in Epidemiology* (STROBE). 

### Lugar

 Unidad de Pediatría del *Hospital Universitari General de Catalunya* (Sant Cugat del Vallés, Barcelona, España) y promoción en *Instagram* (@dospediatrasencasa). 

### Período

El trabajo de campo se realizó entre marzo de 2021 y marzo de 2022.

### Participantes

Padres y tutores de niños que utilizan dispositivos móviles y residen en España.

### Criterios de selección

Padres y tutores de niños de 3 meses a 5 años.

### Definición de la muestra

Muestra por conveniencia y muestreo de bola de nieve.

### Instrumentos de recolección de datos y variables de estudio

 Los datos se obtuvieron mediante un código QR (respuesta rápida) que daba acceso a un cuestionario en línea. El código se incluyó en un folleto informativo sobre el estudio y se entregó a los participantes en la unidad de pediatría del *Hospital Universitari General de Catalunya* (Sant Cugat del Vallés, Barcelona, España) y por difusión en *Instagram* (@dospediatrasencasa). Además, se pidió a los participantes que compartieran el enlace al cuestionario entre los padres de niños de 3 meses a 5 años. 

Los resultados fueron las normas de los padres sobre el uso de dispositivos móviles por parte de los niños y las percepciones sobre la frecuencia de uso y el comportamiento de los niños durante o después del uso de dispositivos móviles.

Se utilizó un cuestionario específico. El cuestionario constó de 10 preguntas en la primera parte y 13 preguntas en la segunda, además de 21 preguntas para abordar aspectos biosociodemográficos del niño y del participante. El cuestionario estaba disponible en línea y podía ser completado por los propios participantes.

 Un conjunto de resultados fueron las normas de los padres con respecto al uso de dispositivos móviles por parte del niño, las percepciones sobre la frecuencia de uso y el comportamiento del niño durante o después de usar el dispositivo móvil ( *smartphones* y/o *tablets* ). Estos resultados fueron evaluados a través de las afirmaciones: 1) Su hijo puede utilizar el *smartphone* o *tablet* sin límite de tiempo; 2) Su hijo puede utilizar su *smartphone* o *tablet* sin restricciones de tiempo los fines de semana; 3) Su hijo sólo puede utilizar su *smartphone* o *tablet* los fines de semana; 4) Su hijo tiene un tiempo al día para utilizar su *smartphone* o *tablet* ; 5) A su hijo le resulta más divertido utilizar su *smartphone* o *tablet* que realizar otras actividades, como deportes, leer, jugar en el recreo u otras; 6) Su hijo usa su *smartphone* o *tablet* por más tiempo del que debería; 7) Su hijo permanece callado cuando deja de usar su *smartphone* o *tablet* ; 8) Su hijo llora o grita cuando deja de usar su dispositivo móvil. El grado de acuerdo con las afirmaciones anteriores se evaluó mediante escalas Likert desde completamente de acuerdo hasta completamente en desacuerdo. También evaluamos el uso de dispositivos móviles para mantener a los niños entretenidos en diferentes ambientes/situaciones, el uso de dispositivos móviles como premio o castigo, y la presencia de los padres mientras el niño utiliza el dispositivo móvil, a través de las afirmaciones: 1) Utiliza *smartphone* o *tablet* para entretener a su hijo o hija en situaciones como restaurantes, citas médicas, transporte público u otras; 2) Utiliza su *smartphone* o *tablet* como premio o castigo, dependiendo del comportamiento de su hijo; 3) Está presente mientras su hijo o hija utiliza su *smartphone* o *tablet* . El grado de acuerdo con las afirmaciones anteriores también se evaluó mediante escalas Likert desde completamente de acuerdo hasta completamente en desacuerdo. El uso diario de *smartphobnes* y *tablets* por parte de los niños se evaluó mediante las preguntas “¿Aproximadamente cuánto tiempo pasa su hijo usando los siguientes dispositivos móviles por día (de lunes a viernes)?” y “¿Aproximadamente cuánto tiempo pasa su hijo usando los siguientes dispositivos móviles los fines de semana (sábado y domingo)?” Las opciones para responder a ambas preguntas incluían categorías de 30 minutos (“0 minutos”, “30 minutos”, “1 hora”, “1 hora y 30 minutos”, “2 horas”, “2 horas y 30 minutos”, “3 horas”, “3 horas y 30 minutos” y “4 horas o más”). El tiempo de uso diario de cada dispositivo se estimó ponderando las respuestas a cada una de las dos preguntas, multiplicándolas por 5 (días escolares entre semana) y 2 (días de fin de semana), respectivamente, sumándolas y dividiéndolas por 7. Para el cálculo se consideró como 4 la categoría de 4 o más horas. 

Variables sociodemográficas asociadas al niño, incluyendo género (masculino/femenino), edad (menor o igual a 2 años/3 a 5 años), tener al menos un hermano (sí/no) y tener al menos un hermano mayor (sí /no); y padres o tutores, incluyendo relación con el niño (madre/padre/otro), edad del entrevistado (menor o igual a 35 años/más de 35 años) y nivel de educación (inferior, primaria y secundaria/universitaria) también fueron recuperados.

### Recolección de datos

 Se utilizaron los datos de referencia del proyecto *Smart Screen Health* , que tiene como objetivo promover el uso saludable de los dispositivos con pantalla en la primera infancia. El cuestionario virtual constaba de 44 preguntas y estuvo disponible en https://www.kenkolab.org/participa/ entre marzo de 2021 y marzo de 2022. 

### Procesamiento y análisis de datos

 El recuento y las proporciones de encuestados que estaban de acuerdo (muy de acuerdo + de acuerdo) con las afirmaciones se estimaron en general y de conformidad con las variables independientes. Se utilizaron las pruebas de chi-cuadrado y exacta de Fisher para explorar la asociación entre el grado de acuerdo y las variables independientes. La normalidad de la variable tiempo de uso del dispositivo móvil se evaluó mediante las pruebas de Kolmogorov -Smirnov y Shapiro-Wilk, así como gráficos QQ. Debido a la no normalidad de los datos, el tiempo de uso del dispositivo móvil se describió con mediana (Me) y rango intercuartil. Se utilizaron las pruebas de Mann-Whitney o Kruskal-Wallis para estimar diferencias significativas en las distribuciones del tiempo de uso de dispositivos móviles entre los encuestados que estaban de acuerdo (completamente de acuerdo + de acuerdo) con las afirmaciones y aquellos que en general no estaban de acuerdo (es decir, niños que usaban un *smartphone* o *tablet* ) y para usuarios exclusivos de *smartphones* y *tablets* . Para realizar los análisis estadísticos se utilizó el programa estadístico R versión 4.0.2. Se realizaron pruebas bilaterales y se estableció un nivel de significancia de 0,05. 

### Aspectos éticos

 El estudio fue evaluado y aprobado por el Comité de Ética en Investigación Clínica del *Grupo Hospitalario Quirónsalud-Catalunya* , el 10/02/2021 (acta núm. 03/2021). Se solicitó el consentimiento informado por escrito después de la lectura de la ficha informativa y antes de otorgar el acceso a la investigación. 

## Resultados

 La muestra final estuvo compuesta por un total de 183 encuestados, que eran padres o tutores de niños que utilizaban *smartphones* o *tablets* , o ambos. Se estima que el 54,1% de los entrevistados eran padres, madres o tutores de hijos varones; 89,1% eran madres y 78,1% tenía estudios universitarios. 

 En cuanto a las normas y restricciones de uso, alrededor del 1,6% (n=3) y el 3,8% (n=7) de los entrevistados coincidieron en que el niño podría utilizar el *smartphone* o la *tablet* sin límite de tiempo y sin limitación de tiempo durante los fines de semana, respectivamente ( Tabla 1 ). La mediana de uso de ambos dispositivos móviles ( *smartphones* y *tablets* ) por parte de los niños fue significativamente mayor entre aquellos que adoptaron una posición favorable a que sus hijos pudieran utilizarlos sin limitaciones de tiempo (Me=103,0 minutos/día), en comparación con los niños cuyos padres no adoptan esta posición. En concreto, este tiempo es significativamente mayor cuando se utiliza únicamente en *smartphones* (Me=77,1 minutos/día). En cambio, presenta menos minutos en aquellos que estipulan que el niño sólo puede utilizar el *smartphone* o la *tablet* los fines de semana (Me=23,6 minutos/día) ( Tabla 2 ). 

 Además, el 18,6% (n=34) de los entrevistados coincidió en que el niño podía utilizar el *smartphone* o la *tablet* sólo durante los fines de semana y el 24,0% (n=44) que el niño tenía un tiempo al día para utilizar el dispositivo ( Tabla 1 ), mientras que el 91,8% (n=168) coincidió en que el padre estuvo presente durante el uso del dispositivo de pantalla ( Tabla 3 ). La mediana general del tiempo de uso fue significativamente mayor para los niños que usaron el dispositivo móvil sin la presencia de su padre en comparación con aquellos que lo usaron con su padre presente (valor p = 0,042) ( Tabla 4 ). En cuanto a las percepciones de uso y frecuencia de uso, el 48,1% (n=88) de los encuestados estuvo de acuerdo en que el niño utilizaba el dispositivo móvil por más tiempo del debido y el 17,5% (n=32) estuvo de acuerdo en que al niño le resultaba más divertido utilizar el *smartphone* o la *tablet* que realizar otras actividades (por ejemplo, hacer deporte, leer, jugar en el recreo) ( Tabla 1 ). Alrededor del 17,5% (n=32) y el 2,7% (n=5) de los encuestados utilizaban el dispositivo móvil para mantener al niño entretenido en diferentes entornos y como premio o castigo ( Tabla 3 ). La mediana general del tiempo de uso fue significativamente mayor en los hijos de padres que utilizaban estas estrategias en comparación con aquellos que no las usaban (valor de p <0,001 y valor de p 0,008, respectivamente) ( Tabla 4 ). 

## Discusión

 Según nuestros resultados, los padres y tutores de los niños de nuestra muestra parecen estar preocupados, en general, por el uso y el tiempo que los niños pasan frente a la pantalla en dispositivos móviles ( *smartphones* y/o *tablets* ). El tiempo frente a una pantalla durante la primera infancia ha sido un problema de salud pública y pediátrica ^(^ 2023 ^)^ . Este puede ser uno de los factores que contribuyen a las disparidades en el desarrollo de la primera infancia. 

 La norma parental de acceso irrestricto de los niños a los dispositivos móviles está asociada a un aumento del tiempo de uso diario, 2,6 veces mayor respecto a aquellos sin acceso libre, lo que puede tener consecuencias para el desarrollo cognitivo, social y emocional de los niños ^(^ 2023 ^)^ . El hecho de que los niños menores de 2 años utilicen dispositivos móviles más de 30 minutos al día, y que este tiempo aumente considerablemente cuando tienen acceso libre, no cumple con ninguna recomendación en materia de tiempo frente a la pantalla y genera preocupación 

**Tabla 1:** - Conteo y porcentajes generales y estratificados de concordancia de padres y tutores con las reglas de uso de dispositivos móviles ( *smartphone/tablet* ) y percepciones sobre la frecuencia de uso y el comportamiento del niño durante o después del uso (n = 183). España, 2021-2022

	**n**	P1* n (%)	**p** ^†^	**(95% IC)** ^‡^	**P2** ^§^ **n (%)**	**p** ^†^	**(95% IC)** ^‡^	**P3** ^||^ **n (%)**	**p** ^†^	**(95% IC)** ^‡^	**P4** ^¶^ **n (%)**	**p** ^†^	**(95% IC)** ^‡^
Total	183	3 (1,6)			7 (3,8)			34 (18,6)			44 (24,0)		
Sexo del niño			0,305	(-0,08 a 0,01)		0,581	(-0,09 a 0,04)		0,421	(-0,08 a 0,01)		0,103	(-0,24 a 0,01)
Masculino	99	3 (3,0)			5 (5,1)			21 (21,2)			29 (29,3)		
Femenino	84	0 (0,0)			2 (2,4)			13 (15,5)			15 (17,9)		
Edad del niño			1,000	(-0,06 a 0,04)		0,801	(-0,04 a 0,09)		0,006	(0,05 a 0,28)		0,874	(-0,15 a 0,11)
Menor o igual a 2 años	100	2 (2,0)			3 (3,0)			11 (11,0)			25 (25,0)		
De 3 a 5 años	83	1 (1,2)			4 (4,8)			23 (27,7)			19 (22,9)		
Hermanos			0,946	(-0,06 a 0,03)		0,099	(-0,13 a -0,0018		0,050	(-0,24 a -0,01)		0,819	(-0,10 a 0,15)
Sí	88	2 (2,3)			6 (6,9)			22 (25,0)			20 (22,7)		
No	95	1 (1,1)			1 (1,1)			12 (12,6)			24 (25,3)		
Hermanos mayores			0,567	(-0,09 a 0,02)		0,089	(-0,15 a 0,00)		0,055	(-0,26 -0,01)		0,813	(-0,11 a 0,15)
Sí	63	2 (3,2)			5 (7,9)			17 (26,9)			14 (22,2)		
No	120	1 (0,8)			2 (1,7)			17 (14,2)			30 (25,0)		
Relación con el niño			1,000	(-0,05 a 0,11)		0,831	(-0,08 a 0,09)		0,727	(-0,14 a 0,24)		1,000	(-0,16 a 0,24)
Madre	163	3 (1,8)			7 (4,3)			30 (18,4)			39 (23,9)		
Padre	17	0 (0,0)	-		0 (0,0)			2 (11,8)	-		4 (23,5)	-	
Otra	3	0 (0,0)			0 (0,0)			2 (66,7)			1 (33,3)		
Edad del encuestado			0,225	(-0,09 a 0,01)		0,398	(-0,10 a 0,03)		0,124	(-0,01 a 0,21)		0,315	(-0,05 a 0,20)
Edad menor o igual a 35 años	89	3 (3,4)			5 (5,6)			12 (13,5)			18 (20,2)		
Mayores de 35 años	94	0 (0,0)			2 (2,1)			22 (23,4)			26 (27,7)		
Nivel de escolaridad			0,234	(-0,15 a 0,09)		1,000	(-0,12 a 0,05)		0,158	-0,28 a 0,03)		0,639	(-0,11 a 0,18)
Inferior, primario o secundario	40	2 (5,0)			2 (5,0)			11 (27,5)			8 (20,0)		
Estudios universitarios	143	1 (0,7)			5 (3,5)			23 (16,1)			36 (25,2)		
	**n**	P5** n (%)	**p** ^†^	**(95% IC)** ^‡^	**P6** ^††^ **n (%)**	**p** ^†^	**(95% IC)** ^‡^	**P7** ^‡‡^ **n (%)**	**p** ^†^	**(95% IC)** ^‡^	**P8** ^§§^ **n (%)**	**p** ^†^	**(95% IC)** ^‡^
Total	183	32 (17,5)			88 (48,1)			100 (54,6)			54 (29,5)		
Sexo del niño			0,941	(-0,13 a 0,10)		0,080	(-0,28 a 0,01)		1,000	(-0,14 a 0,15)		0,456	(-0,19 a 0,07)
Masculino	99	18 (18,2)			54 (54,6)			54 (54,6)			32 (32,3)		
Femenino	84	14 (16,7)			34 (40,5)			46 (54,8)			22 (26,2)		
Edad del niño			0,431	(-0,17 a 0,06)		0,286	(-0,06 a 0,23)		0,124	(-0,02 a 0,27)		0,051	(-0,27 a -0,01)
Menor o igual a 2 años	100	20 (20,0)			44 (44,0)			49 (49,00)			36 (36,0)		
De 3 a 5 años	83	12 (14,5)			44 (53,0)			51 (61,5)			18 (21,7)		
Hermanos			0,410	(-0,17 a 0,05)		0,067	(-0,29 a -0,00)		0,056	(-0,29 a -0,01)		0,862	(-0,16 a 0,11)
Sí	88	18 (20,5)			49 (55,7)			55 (62,5)			27 (30,7)		
No	95	14 (14,7)			39 (41,1)			45 (47,4)			27 (28,4)		
Hermanos mayores			0,543	(-0,18 a 0,07)		0,053	(-0,31 -0,01)		0,202	(-0,26 a 0,04)		1,000	(-0,15 a 0,13)
Sí	63	13 (20,6)			37 (58,7)			39 (61,9)			19 (30,2)		
No	120	19 (15,8)			51 (42,5)			61 (50,8)			35 (29,2)		
Relación con el niño			0,288	(-0,04 a 0,37)		0,381	(-0,35 a 0,09)		0,014	(-0,46 a -0,04)		0,823	(-0,18 a 0,24)
Madre	163	26 (15,9)			81 (49,7)			94 (57,7)			48 (29,5)		
Padre	17	5 (29,4)	-		6 (35,3)	-		4 (23,5)	-		6 (35,3)	-	
Otra	3	1 (33,3)			1 (33,3)			2 (66,7)			0 (0,0)		
Edad del encuestado			0,678	(-0,08 a 0,15)		1,000	(-0,15 a 0,14)		0,579	(-0,20 a 0,09)		0,567	(-0,08 a 0,18)
Edad menor o igual a 35 años	89	14 (15,7)			43 (48,3)			51 (57,3)			24 (26,9)		
Mayores de 35 años	94	18 (19,2)			45 (47,9)			49 (52,1)			30 (31,9)		
Nivel de escolaridad			0,811	(-0,19 a 0,09)		0,924	(-0,20 a 0,15)		0,625	(-0,11 a 0,23)		0,366	(-0,07 a 0,23)
Inferior, primario o secundario	40	8 (20,0)			20 (50,0)			20 (50,0)			9 (22,5)		
Estudios universitarios	143	24 (17,8)			68 (47,6)			80 (55,9)			45 (31,5)		

* P1 = Su hijo puede utilizar el *smartphone* o la *tablet* sin límite de tiempo

^†^ p = valor p (Cada valor p proviene del valor de chi -cuadrado según la prueba de Fisher)

^‡^ IC95% = Intervalo de confianza del 95%

^§^ P2 = Su hijo puede utilizar su *smartphone* o *tablet* sin restricciones de tiempo los fines de semana

^||^ P3 = Su hijo solo puede utilizar el *smartphone* o *tablet* los fines de semana

^¶^ P4 = Su hijo tiene un tiempo al día para usar su *smartphone o tablet* ?

** P5 = A su hijo le resulta más divertido usar su *smartphone* o *tablet* que realizar otras actividades, como deportes, leer, jugar en el recreo u otras

^††^ P6 = Su hijo usa su *smartphone* o *tablet* por más tiempo del que debería

^‡‡^ P7 = Su hijo permanece callado cuando deja de usar su *smartphone o tablet*

^§§^ P8 = Su hijo llora o grita cuando deja de usar su *smartphone* o *tablet*

**Tabla 2:** - Medianas y cuartiles de tiempo de uso (en minutos por día) generales y estratificados por dispositivo en niños, según la concordancia con las normas de uso de dispositivos móviles y las percepciones de frecuencia de uso y comportamiento infantil durante o después del uso, según padres y tutores (n = 183). España, 2021-2022

	***Smartphone* y/o *tablet* (n=183)**	**Solo *Smartphone* (n=139)**	**Solo *tablet* (n=80)**
	Mediana (RIQ*)	Mediana (RIQ*)	Mediana (RIQ*)
P1 ^†^			
Estoy de acuerdo ^‡^	103,0 (90,0-111,0)	77,1 (68,6-90,0)	60,0 (60,0-60,0)
Rest ^§^	30,0 (17,1-51,4)	30,0 (8,57-30,0)	30,0 (17,1-60,0)
p ^||^	0.010	0.005	0.912
P2 ^¶^			
Estoy de acuerdo ^‡^	55,7 (27,9-90,0)	60,0 (30,0-77,1)	17,1 (8,57-47,1)
Rest ^§^	30,0 (17,1-51,4)	30,0 (8,57-30,0)	30,0 (17,1-62,1)
p ^||^	0.164	0.402	0.305
P3**			
Estoy de acuerdo ^‡^	23,6 (8,6-54,6)	17,1 (8,57-34,3)	21,4 (8,6-55,7)
Rest ^§^	30,0 (21,4-55,7)	30,0 (17,1-38,6)	30,0 (24,6-65,4)
p ^||^	0.102	0.026	0,167
P4			
Estoy de acuerdo ^‡^	30,0 (8,6-55,7)	27,9 (8,6-30,0)	30,0 (23,6-60,0)
Rest ^§^	30,0 (19,3-53,6)	30,0 (17,1-38,6)	30,0 (17,1-60,0)
p ^||^	0.527	0.012	0.215
P5 ^‡‡^			
Estoy de acuerdo ^‡^	34,3 (17,1-63,2)	30,0 (8,57-38,6)	34,3 (25,7-58,9)
Rest ^§^	30,0 (17,1-40,7)	30,0 (8,57-30,0)	30,0 (10,7-60,0)
p ^||^	0.256	0,773	0.101
P6 ^§§^			
Estoy de acuerdo ^‡^	38,6 (30,0-66,4)	30,0 (21,43-60,0)	34,3 (17,1-73,9)
Rest ^§^	30,0 (8,6-30,0)	30,0 (8,57-30,0)	30,0 (8,57-40,7)
p ^||^	< 0,001	0.005	0.038
P7 ^||||^			
Estoy de acuerdo ^‡^	30,0 (20,4-60,0)	30,0 (8,57-38,6)	30,0 (22,5-71,8)
Rest ^§^	30,0 (17,1-38,6)	30,0 (8,57-30,0)	30,0 (8,57-50,4)
p ^||^	0,047	0,627	0.024
P8			
Estoy de acuerdo ^‡^	30,0 (17,1-48,2)	30,0 (17,1-38,6)	30,0 (8,57-60,0)
Rest ^§^	30,0 (17,1-55,7)	30,0 (8,57-30,0)	30,0 (24,6-61,1)
p ^||^	0.970	0.702	0,277

* RIQ = Rango intercuartil

^†^ P1 = Su hijo puede utilizar el *smartphone* o la *tablet* sin límite de tiempo

^‡^ De acuerdo = Estoy completamente de acuerdo + Estoy de acuerdo

^§^ Rest = Ni de acuerdo ni en desacuerdo + En desacuerdo + Completamente en desacuerdo

^||^ p = valor p (Cada valor de p proviene de la prueba U de Mann-Whitney )

^¶^ P2 = Su hijo puede usar su *smartphone* o *tablet* sin restricciones de tiempo los fines de semana

** P3 = Su hijo solo puede usar su *smartphone* o *tablet* los fines de semana

^††^ P4 = Su hijo tiene un tiempo al día para usar su *smartphone o Tablet*

^‡‡^ P5 = A su hijo le resulta más divertido usar su *smartphone* o *tablet* que realizar otras actividades, como deportes, leer, jugar en el recreo u otras

^§§^ P6 = Su hijo usa su *smartphone* o *tablet* por más tiempo del que debería

^||||^ P7 = Su hijo permanece callado cuando deja de usar su *smartphone* o *tablet*

^¶¶^ P8 = Su hijo llora o grita cuando deja de usar su *smartphone* o *tablet*

 sobre el impacto de la exposición temprana a la tecnología en el desarrollo infantil ^(^ 2023 ^)^ . Una proporción muy baja de padres utiliza su *smartphone* o *tablet* como premio o castigo, con un uso medio de 2 horas al día, dependiendo del comportamiento del niño. El hecho de que la mayoría no recurra a esta estrategia disciplinaria es tranquilizador, pero es preocupante aquellos que sí la utilizan para no cumplir las recomendaciones. 

La presencia de los padres en el uso de dispositivos se asoció con tener un hermano, pero no con tener un

**Tabla 3:** - Conteo y porcentajes de concordancia con estrategias de uso de dispositivos móviles ( *smart phone/tablet* ) para mantener a los niños entretenidos en diferentes entornos, utilizando dispositivos móviles como premio o castigo, entre padres y tutores (n = 183). España, 2021-2022

	**n**	Uso del dispositivo móvil como entretenimiento en diferentes entornos* n (%)	**p** ^†^	**(IC del 95%)** ^‡^	**Uso del dispositivo móvil como premio/castigo n (%)**	**p** ^†^	**(IC del 95%)** ^‡^	**Presencia de los padres durante el uso del dispositivo móvil n (%)**	**p** ^†^	**(IC del 95%)** ^‡^
Total	183	32 (17,5)			5 (2,7)			168 (91,8)		
Sexo del niño			0.213	(-0,19 a 0,03)		1.000	(-0,06 a 0,05)		0.453	(-0,04 a 0,12)
Masculino	99	21 (21,2)			3 (3.0)			89 (89,0)		
Femenino	84	11 (13.1)			2 (2.4)			79 (94,0)		
Edad del niño			0,437	(-0,06 a 0,17)		0.832	(-0,04 a 0,08)		0.144	(-0,16 a 0,01)
Edad menor o igual a 2 años	100	15 (15,0)			2 (2.0)			95 (95,0)		
De 3 a 5 años	83	17 (20,5)			3 (3,6)			73 (88,0)		
Hermanos			0.410	(-0,17 a 0,05)		0.320	(-0,10 a 0,02)		0.020	(0,03 a 0,19)
Sí	88	18 (20,5)			4 (4,5)			76 (86,4)		
No	95	14 (14,7)			1 (1,1)			92 (96,8)		
Hermanos mayores			0.153	(-0,23 a 0,02)		0,089	(-0,14 a -0,00)		1.000	(-0,09 a 0,09)
Sí	63	15 (23,8)			4 (6,3)			58 (92,1)		
No	120	17 (14,2)			1 (0,8)			110 (91,7)		
Relación con el niño			0.750	(-0,12 a 0,25)		1.000	(-0,07 a 0,10)		0.004	(-0,41 a -0,03)
Madre	163	28 (17,2)			5 (3.1)			153 (93,9)		
Padre	17	4 (23,5)			0 (0,0)			12 (70,6)		
Otra	3	0 (0,0)			0 (0,0)			3 (100,0)		
Edad del encuestado			0.006	(-0,27 a -0,05)		0.060	(-0,12 a -0,01)		1.000	(-0,09 a 0,08)
Menor o igual a 35 años	89	23 (25,8)			5 (5,6)			82 (92,1)		
Mayores de35 años	94	9 (9,6)			0 (0,0)			86 (91,5)		
Nivel de escolaridad			0.238	(-0,26 a 0,04)		1.000	(-0,09 a 0,05)		0,885	(-0,07 a 0,15)
Inferior, primaria y secundario	40	10 (25,0)			1 (2,5)			36 (90,0)		
Estudios universitarios	143	22 (15,4)			4 (2,8)			132 (92,3)		

* Diferentes Situaciones = Restaurantes, citas médicas, transporte público u otros

^†^ p = valor p (cada valor p proviene del valor de chi-cuadrado según la prueba de Fisher)

^‡^ IC del 95% = Intervalo de confianza del 95%

**Tabla 4:** - Medianas y cuartiles de tiempo de uso (en minutos por día) en niños, según el dispositivo móvil y estrategias diarias de uso de dispositivos móviles, uso como premio/castigo y presencia de los padres, en padres y tutores (n = 183). España, 2021-2022

	***Smartphone* y *tablet***	**Solo s *martphone***	**Solo *tablet***
	Mediana (RIQ*)	Mediana (RIQ*)	Mediana (RIQ*)
Usar un dispositivo móvil como entretenimiento en diferentes situaciones ^†^			
Estoy de acuerdo ^‡^	49,3 (30,0-80,4)	30,0 (17,1-47,1)	53,6 (28,9-90,0)
Rest ^§^	30,0 (17,1-38,6)	30,0 (8,57-30,0)	30,0 (10,7-55,7)
p ^||^	< 0,001	< 0,001	0.510
Uso del dispositivo móvil como recompensa/castigo			
Estoy de acuerdo ^‡^	120,0 (55,7-163,0)	30,0 (30,0-60,0)	81,4 (51,4-114,0)
Rest ^§^	30,0 (17,1-50,4)	30 (8,57-36,4)	30,0 (15,0-60,0)
p ^||^	0.008	0.051	0.020
Presencia de los padres durante el uso del dispositivo móvil			
Estoy de acuerdo ^‡^	30,0 (17,1-48,2)	30,0 (8,57-30,0)	30,0 (8,57-60,0)
Rest ^§^	47,1 (23,6-101,0)	30,0 (8,57-60,0)	38,6 (25,7-75,0)
p ^||^	0,042	0,668	0.005

* RIQ = Rango intercuartil

^†^ Diferentes Situaciones = Restaurantes, citas médicas, transporte público u otros

^‡^ De acuerdo = Estoy completamente de acuerdo +De acuerdo

^§^ Rest = Ni de acuerdo ni en desacuerdo + En desacuerdo + Completamente en desacuerdo

^||^ p = valor p (cada valor p proviene de la prueba U de Mann-Whitney)

 hermano mayor. Otro estudio indicó que la presencia de un hermano y un espacio menos personal en la sala de estar eran predictores significativos del tiempo que los niños pasan frente a la pantalla ^(^ 2021 ^)^ . Gestionar el uso del *smartphone* guiado por el control parental no es muy eficaz y empeora la adicción al *smartphone* , teniendo en cuenta que los padres que pasan más tiempo delante de la pantalla perciben con menos preocupación el tiempo que pasan sus hijos frente a la pantalla ^(^ 2019 ^,^ 2021 ^)^ . Esto sugiere que los factores socioculturales y ambientales en el hogar y en la comunidad, además de los factores conductuales de los padres, como el conocimiento sobre los efectos de los dispositivos móviles en la salud, pueden favorecer su uso por parte de los niños. Las instituciones de salud y el personal de salud, en especial los enfermeros, deben asumir el desafío de concientizar a niños y adolescentes sobre el uso responsable de los dispositivos móviles ^(^ 2021 ^)^ . Más aún si tenemos en cuenta que, a medida que los niños crecen, también aumenta el nivel de autonomía e independencia en el uso de dispositivos móviles, lo que coincide con los resultados de este estudio ^(^ 2017 ^,^ 2024 ^)^ . 

 Los padres de más edad, que informan que sus hijos pasan más tiempo usando dispositivos móviles, pueden enfrentar desafíos adicionales en lo que se refiere a establecer límites y promover un equilibrio entre el tiempo frente a la pantalla y otras actividades, ya que se ha demostrado que cuanto mayores son los padres y cuanto más tiempo pasan viendo dispositivos móviles, mayor es el tiempo que pasan sus hijos frente a la pantalla ^(^ 2023 ^)^ . Las diferencias en el tiempo dedicado a los dispositivos móviles pueden reflejar diferentes actitudes y percepciones hacia la tecnología entre los padres mayores, quienes pueden tener una visión más permisiva o valorar más los beneficios potenciales de la tecnología. Por otro lado, los padres más jóvenes pueden ser más cautelosos o restrictivos con respecto al tiempo que pasan frente a los dispositivos móviles. Afortunadamente, la mayoría de los padres (91,8%) están presentes mientras sus hijos utilizan dispositivos móviles, lo que indica una supervisión parental activa y permisividad durante el uso de dispositivos móviles. Encontramos asociaciones, como el uso de dispositivos móviles exclusivamente los fines de semana, con la edad del niño y la relación con él (madre o padre), lo que se explica porque la sociedad española ha experimentado cambios drásticos en el estilo de vida en las últimas décadas. 

 Otra asociación fue la percepción de que el niño permanece tranquilo cuando deja de usar el dispositivo móvil y la relación con el niño (madre o padre), lo que apunta a la idea de que algunos padres creen que el uso puede tener un impacto positivo en el comportamiento de sus hijos. Las asociaciones muestran que este nuevo determinante de la salud requiere intervenciones diferentes, en virtud de los riesgos que implica. Casi una cuarta parte de los niños españoles de 1 a 14 años que superaron los 120 minutos diarios de pantalla en actividades de ocio tuvieron una reducción en la duración del sueño ^(^ 2021a ^)^ . 

 Hasta la fecha, los estudios no se han centrado en estudiar las normas y percepciones de padres y tutores en Europa sobre el uso de dispositivos con pantalla. Casi todos se encuentran en países asiáticos y anglosajones ^(^ 2023 ^,^ 2021 ^,^ 2023 ^,^ 2011 ^)^ . Hasta donde sabemos, este es uno de los primeros estudios que describe las normas y percepciones de los padres de niños que utilizan dispositivos móviles, dejando atrás el paradigma televisivo. La inclusión de padres, madres y tutores, así como la variabilidad en los niveles educativos, ofrece una perspectiva amplia y permite resultados más generales. La combinación de datos numéricos y respuestas perceptuales proporciona una comprensión más completa del uso de los dispositivos móviles por parte de los niños. El estudio aborda la cuestión del acceso y uso de los dispositivos móviles por parte de los niños, que es relevante hoy en día y puede tener implicaciones para la crianza y el desarrollo infantil. 

 En el futuro, se espera un drástico aumento en el uso de dispositivos móviles por parte de los niños. Esto resalta la importancia de brindar educación y apoyo a los padres sobre el uso de pantallas durante los primeros años de vida, considerando las realidades de la sociedad actual ^(^ 2023 ^)^ . Existe evidencia de que el enfermero puede intervenir en el contexto de la educación de los padres para cumplir con las recomendaciones sobre el tiempo de pantalla en los niños, teniendo en cuenta que este es un determinante social de la salud en nuestros tiempos ^(^ 2023 ^,^ 2022 ^)^ . La colaboración entre investigadores, formuladores de políticas y padres es esencial para actualizar las recomendaciones a pediatras y enfermeros para administrar adecuadamente el tiempo frente a una pantalla durante la primera infancia ^(^ 2021 ^,^ 2023 ^,^ 2024 ^)^ . 

 El presente estudio tiene algunas limitaciones. En primer lugar, la selección de participantes puede haber generado sesgos y limitado la representatividad de la muestra. Además, no preguntamos sobre el uso de dispositivos móviles por parte de los padres, lo que podría influir en el tiempo de uso de sus hijos ^(^ 2023 ^,^ 2019 ^)^ . No se proporciona información sobre el proceso de desarrollo y validación del cuestionario, lo que genera dudas sobre la confiabilidad y validez de las medidas utilizadas. La evaluación del tiempo de uso en intervalos de 30 minutos puede ser inexacta y puede depender de la memoria de los participantes. En la última categoría, limitamos el tiempo de uso a un máximo de 4 horas. Por lo tanto, si hay niños que han consumido más de este tiempo, no los tenemos en cuenta. Este estudio no verificó los aspectos cualitativos del fenómeno y fue un autoinforme del participante sobre el tiempo de uso de dispositivos móviles en los niños, considerando la declaración del cuidador; sin embargo, esto apoya la idea de realizar más investigaciones sobre este aspecto. 

## Conclusión

Las reglas de los padres influyen en el tiempo que los niños pasan en los dispositivos móviles. Los permisos ilimitados dan como resultado más tiempo frente a la pantalla, con posibles efectos en el desarrollo de los niños. La supervisión de los padres también es importante; sin ella, los niños pasan más tiempo frente a las pantallas. Muchos padres admiten que sus hijos superan los límites recomendados y prefieren los dispositivos a otras actividades. Estas percepciones influyen en el tiempo frente a la pantalla y señalan la necesidad de supervisión parental y educación temprana. Los profesionales de la salud, como los enfermeros, pueden desempeñar un papel esencial en dos áreas principales: educar a los padres para que cumplan las recomendaciones y promover patrones saludables de tiempo frente a la pantalla en niños y adolescentes.

# Translation: Normas e percepções dos pais com relação ao uso de dispositivos móveis por crianças menores de cinco anos *

**DOI:** 10.1590/1518-8345.7137.4363

**Published:** 2024-10-09

**Authors:** David San-Martín-Roldán, Adrián González-Marrón, Sonia de-Paz-Cantos, Cristina Lidón-Moyano, Ana Díez-Izquierdo, Jose M Martínez-Sánchez

**Affiliations:** ^1^ Universitat Internacional de Catalunya, Department of Basic Sciences, Barcelona, Catalonia, Espanha.; ^2^ Universidad Diego Portales, Facultad de Salud y Odontología, Santiago, Región Metropolitana, Chile.; ^3^ Kenko Lab, Centro de estudios del uso saludable de pantallas, Barcelona, Catalonia, Espanha.; ^4^ Instituto de Efectividad Clínica y Sanitaria, Departamento de Evaluación de Tecnologías Sanitarias (ETS) y Economía de la Salud, Buenos Aires, Argentina.; ^5^ Hospital Universitario Vall d’Hebron, Sección de Alergia Pediátrica, Neumología Pediátrica y Fibrosis Quística Infantil, Barcelona, Catalonia, Espanha.; ^6^ Universidad de Extremadura, Centro Universitario de Mérida, Group of Evaluation of Health Determinants and Health Policies, Mérida, Badajoz, Espanha.

**Keywords:** Dispositivo Móvel, Tablet, Smartphone, Crianças Pré-Escolares, Tempo de Tela, Percepção

## Abstract

descrever as normas e as percepções de uma amostra de pais e responsáveis espanhóis sobre o uso de dispositivos móveis ( *smartphone* e/ou *tablet* ) por seus filhos e sua associação com esse uso.

estudo quantitativo, analítico, transversal, *online* e exploratório foi realizado com uma amostra não probabilística de pais ou responsáveis por crianças que usam *smartphones* ou *tablets* . O questionário virtual consistia em 44 perguntas e estava disponível em https://www.kenkolab.org/participa/ .

o número total de participantes (pais ou responsáveis) foi de 183 pessoas. Observou-se que 26,2% dos pais admitiram que seus filhos tinham acesso livre e irrestrito a *smartphones* ou *tablets* . Foram identificadas associações entre o tempo gasto com o uso de dispositivos móveis e as atitudes dos pais, como a permissão sem limitação de tempo e o uso como recompensa ou punição. Aproximadamente metade dos entrevistados acredita que seus filhos usam o dispositivo móvel por mais tempo do que o recomendado.

a maioria dos pais exerce algum controle sobre o uso de dispositivos por seus filhos. Agir de acordo com as normas e percepções dos pais é vital para o sucesso na abordagem do tempo que as crianças passam em dispositivos móveis. A enfermagem comunitária pode ser um meio de educação sobre hábitos saudáveis de consumo de tela na primeira infância.

## Destaques

**(1)** A maioria dos pais está preocupada com o tempo que seus filhos passam usando dispositivos móveis.

**(2)** O acesso irrestrito está associado ao aumento do uso de dispositivos móveis por crianças.

**(3)** Os pais estão preocupados com a exposição precoce das crianças aos dispositivos móveis.

**(4)** A presença dos pais influencia o tempo de uso de dispositivos móveis das crianças.

**(5)** Necessidade de educação e colaboração.

## Introdução

 A oferta de aplicativos para dispositivos móveis ( *smartphone* e/ou *tablet* ) aumentou com o uso, sendo que a porcentagem de crianças de três a quatro anos que usam um dispositivo móvel em casa cresceu de 28% em 2013 para 63% em 2019, de acordo com o *UK Office of Communications* ^(^ 2021 ^)^ . Nesse sentido, os dispositivos móveis estão substituindo os computadores e agora são uma parte central da vida das crianças ^(^ 2021 ^-^ 2021 ^)^ . No caso de crianças de zero a oito anos, 33% do tempo de tela é gasto na frente de um dispositivo móvel nos Estados Unidos ^(^ 2023 ^)^ . Na Espanha, mais de 65% da população com idade entre dez e 15 anos tem seu próprio celular, o que significa uma integração precoce na vida diária das crianças espanholas ^(^ 2021 ^,^ 2021b ^)^ . 

 As evidências sobre os riscos e benefícios do uso de dispositivos móveis em crianças são contraditórias ^(^ 2020 ^)^ . Entre os riscos, seu uso regular está associado a efeitos prejudiciais em seu desenvolvimento físico, neurológico, psicológico e social ^(^ 2021 ^,^ 2023 ^)^ . Há muitas evidências de que o aumento do uso de dispositivos móveis em crianças está associado à redução da duração do sono, atraso na hora de dormir, problemas de sono, má qualidade do sono ^(^ 2024 ^)^ e má nutrição ^(^ 2021b ^,^ 2020 ^)^ . Além disso, quando combinado com baixos níveis de atividade física durante a infância, o tempo de tela aumenta o risco de obesidade ^(^ 2023 ^,^ 2011 ^)^ e depressão ^(^ 2021 ^-^ 2020 ^)^ . Por outro lado, o uso de dispositivos móveis pode ter efeitos positivos se o conteúdo apropriado for absorvido na presença dos pais ou cuidadores da criança ^(^ 2023 ^,^ 2020 ^)^ . Esse é o caso de programas educacionais de alta qualidade destinados a oferecer oportunidades de aprendizado, mas somente para crianças com mais de dois anos de idade ^(^ 2023 ^,^ 2017 ^)^ . Além disso, assistir a uma quantidade moderada de programas de alta qualidade, por exemplo, “Vila Sésamo”, melhora os resultados cognitivos, sociais e de desempenho escolar ^(^ 2019 ^)^ . Os *smartphones* podem até ser um recurso para manter a socialização estando ao ar livre ^(^ 2021 ^)^ . 

 Os pais desempenham um papel fundamental no uso e no tempo gasto pelos filhos com dispositivos móveis (tempo de tela) ^(^ 2023 ^)^ . Entretanto, os pais geralmente não se preocupam com a quantidade de tempo, mas sim com o conteúdo ^(^ 2023 ^)^ . Além disso, evidências indicam que os pais apontam vários benefícios do uso de dispositivos móveis por seus filhos: como ferramenta educacional e de entretenimento, como dispositivo para auxiliar na hora de dormir e também para promover o vínculo familiar e a atividade física, por meio de brincadeiras ativas ^(^ 2023 ^)^ . Além disso, parece haver diferenças nas normas, percepções, preocupações e abordagens usadas pelos pais para gerenciar o tempo de tela dos filhos. Os pais que passam mais tempo usando dispositivos de tela percebem o tempo de tela de seus filhos com menos preocupação ^(^ 2019 ^)^ . Os pais que têm uma visão positiva da tecnologia digital não apenas baixam aplicativos para seus filhos com mais frequência do que os outros pais, mas também tentam comprar aplicativos em vez de usar apenas aplicativos gratuitos. Além disso, esses pais tentam equipar seus filhos com pelo menos um tipo de dispositivo móvel inteligente ^(^ 2021 ^)^ . 

 O uso inadequado de dispositivos móveis é um importante problema de saúde pública na população pediátrica. O papel das profissões de saúde, especialmente da enfermagem comunitária, com intervenções educacionais, tem se mostrado eficaz na redução do tempo de tela em crianças ^(^ 2023 ^)^ . Dado o papel fundamental desempenhado pelos pais, o objetivo deste estudo foi descrever as normas e percepções de uma amostra de pais e responsáveis espanhóis sobre o uso de dispositivos móveis ( *smartphones* e/ou *tablets* ) pelos filhos e sua associação com esse uso. 

## Método

### Desenho do estudo

 Estudo observacional, analítico e transversal realizado de acordo com as diretrizes do *Strengthening the Reporting of Observational Studies in Epidemiology* (STROBE). 

### Local

 Unidade de Pediatria do *Hospital Universitari General de Catalunya* (Sant Cugat del Vallés, Barcelona, Espanha) e por meio de divulgação no *Instagram* (@dospediatrasencasa). 

### Período

O trabalho de campo foi realizado entre março de 2021 e março de 2022.

### Participantes

Pais e responsáveis por crianças que usam dispositivos móveis e moram na Espanha.

### Critérios de seleção

Pais e responsáveis por crianças de três meses a cinco anos de idade.

### Definição da amostra

Amostragem por conveniência e bola de neve.

### Instrumentos de coleta de dados e variáveis do estudo

 Os dados foram obtidos por meio de um código QR (resposta rápida) que dava acesso a um questionário *online* . O código estava incluído em um folheto informativo sobre o estudo e foi entregue aos participantes na unidade de pediatria do *Hospital Universitari General de Catalunya* (Sant Cugat del Vallés, Barcelona, Espanha) e por meio de transmissão no Instagram (@dospediatrasencasa). Além disso, foi solicitado aos participantes que divulgassem o *link* para o questionário entre os pais de crianças de três meses a cinco anos de idade. 

Os resultados foram as normas dos pais sobre o uso de dispositivos móveis pelas crianças e as percepções sobre a frequência de uso e o comportamento das crianças durante ou após o uso do dispositivo móvel.

 Foi usado um questionário específico. O questionário consistia em dez perguntas na primeira parte e 13 perguntas na segunda, além de 21 perguntas para abordar aspectos bio-sociodemográficos da criança e do participante. O questionário estava disponível *online* e podia ser preenchido pelo próprio participante. 

 Um conjunto de resultados foram as normas dos pais sobre o uso de dispositivos móveis pela criança, as percepções sobre a frequência de uso e o comportamento da criança durante ou após o uso do dispositivo móvel ( *smartphone* e/ou *tablet* ). Esses resultados foram avaliados por meio das declarações: 1) Seu filho pode usar o *smartphone* ou *tablet* sem limitação de tempo; 2) Seu filho pode usar o *smartphone* ou *tablet* sem limitação de tempo nos fins de semana; 3) Seu filho pode usar o *smartphone* ou *tablet* somente nos fins de semana; 4) Seu filho tem um horário por dia para usar o *smartphone* ou *tablet* ; 5) Seu filho acha mais divertido usar o *smartphone* ou *tablet* do que fazer outras atividades, como esportes, leitura, brincar no recreio ou outras atividades; 6) Seu filho usa o *smartphone* ou *tablet* por mais tempo do que deveria; 7) Seu filho fica quieto quando deixa de usar o *smartphone* ou *tablet* ; 8) Seu filho chora ou grita quando deixa de usar o dispositivo móvel. O grau de concordância com as afirmações acima foi avaliado por escala tipo *Likert* variando de concordo totalmente a discordo totalmente. Também avaliamos o uso de dispositivos móveis para manter as crianças entretidas em diferentes ambientes/situações, o uso de dispositivos móveis como recompensa ou punição e a presença dos pais enquanto a criança usa o dispositivo móvel, por meio das declarações: 1) Você usa o *smartphone* ou *tablet* para entreter seu filho ou filha em situações como restaurantes, consultas médicas, transporte público ou outras; 2) Você usa o *smartphone* ou *tablet* como recompensa ou punição, dependendo do comportamento do seu filho; 3) Você está presente enquanto seu filho ou filha está usando o *smartphone* ou *tablet* . O grau de concordância com as afirmações acima também foi avaliado por escala tipo *Likert* variando de concordo totalmente a discordo totalmente. O uso diário de *smartphone* e *tablet* das crianças foi avaliado por meio das perguntas “Aproximadamente, quanto tempo seu filho passa usando os seguintes dispositivos móveis por dia (de segunda a sexta-feira)?” e “Aproximadamente, quanto tempo seu filho passa usando os seguintes dispositivos móveis nos fins de semana (sábado e domingo)?”. As opções para responder a ambas as perguntas incluíam categorias de 30 minutos (“0 minutos”, “30 minutos”, “1 hora”, “1 hora e 30 minutos”, “2 horas”, “2 horas e 30 minutos”, “3 horas”, “3 horas e 30 minutos” e “4 horas ou mais”). O tempo de uso diário de cada dispositivo foi estimado pela ponderação das respostas a cada uma das duas perguntas, multiplicando-as por 5 (dias letivos da semana) e 2 (dias de fim de semana), respectivamente, somando-as e dividindo-as por 7. Para esse cálculo, a categoria 4 ou mais horas foi considerada como 4. 

Variáveis sociodemográficas associadas à criança, incluindo gênero (masculino/feminino), idade (menor ou igual a 2 anos/ de 3 a 5 anos), ter pelo menos um irmão (sim/não) e ter pelo menos um irmão mais velho (sim/não); e aos pais ou responsáveis, incluindo relacionamento com a criança (mãe/pai/outro), idade do entrevistado (menor ou igual a 35 anos/mais de 35 anos) e nível de escolaridade (inferior, primário e secundário/universitário) também foram recuperadas.

### Coleta de dados

 Foram usados dados de linha de base do projeto *Smart Screen Health* , que visa promover o uso saudável de dispositivos de tela na primeira infância. O questionário virtual consistia em 44 perguntas e estava disponível em https://www.kenkolab.org/participa/ entre março de 2021 e março de 2022. 

### Tratamento e análise de dados

 As contagens e proporções de entrevistados que concordam (concordam fortemente + concordam) com as afirmações foram estimadas de forma geral e de acordo com as variáveis independentes. Os testes de qui-quadrado e exato de Fisher foram usados para explorar a associação entre o grau de concordância e as variáveis independentes. A normalidade da variável tempo de uso do dispositivo móvel foi avaliada por meio dos testes de Kolmogorov-Smirnov e Shapiro-Wilk, bem como dos gráficos Q-Q. Devido à não normalidade dos dados, o tempo de uso do dispositivo móvel foi descrito com mediana (Me) e intervalo interquartil. Os testes de Mann-Whitney ou Kruskal-Wallis foram usados para estimar diferenças significativas nas distribuições do tempo de uso de dispositivos móveis entre os entrevistados que concordaram (concordam fortemente + concordam) com as afirmações e aqueles que não concordaram, em geral (ou seja, crianças que usaram *smartphone* ou *tablet* ) e para usuários exclusivos de *smartphone* e *tablet* . Para realizar as análises estatísticas, foi usado o programa estatístico R versão 4.0.2. Foram realizados testes bicaudais e foi estabelecido um nível de significância de 0,05. 

### Aspectos éticos

 O estudo foi avaliado e aprovado pelo Comitê de Ética em Pesquisa Clínica do *Grupo Hospitalario Quirónsalud-Catalunya* , em 10/02/2021 (ata nº 03/2021). O consentimento informado por escrito foi solicitado após a leitura do documento de informações e antes de dar acesso à pesquisa. 

## Resultados

 A amostra final consistiu em um total de 183 entrevistados, que eram pais ou responsáveis por crianças que usavam *smartphone* s ou *tablet* s, ou ambos. Estima-se que 54,1% dos entrevistados eram pais, mães ou responsáveis por crianças do sexo masculino; 89,1% eram mães e 78,1% tinham estudos universitários. 

 Com relação às regras e restrições de uso, cerca de 1,6% (n=3) e 3,8% (n=7) dos entrevistados concordaram que a criança poderia usar o *smartphone* ou *tablet* sem limitação de tempo e sem limitação de tempo nos fins de semana, respectivamente (Tabela 1). A mediana de uso de ambos os dispositivos móveis ( *smartphone* e *tablet* ) pelas crianças foi significativamente maior naqueles que adotam uma posição a favor de que seus filhos possam usá-los sem limitação de tempo (Me=103,0 minutos/dia), em comparação com as crianças cujos pais não adotam essa posição. Especificamente, esse tempo é significativamente maior quando o uso é apenas em *smartphone* s (Me=77,1 minutos/dia). Em contrapartida, apresenta minutos menores naqueles que estipulam que o filho só pode usar o *smartphone* ou *tablet* nos fins de semana (Me=23,6 minutos/dia) (Tabela 2). 

 Além disso, 18,6% (n=34) dos entrevistados concordaram que a criança podia usar o *smartphone* ou *tablet* somente nos fins de semana e 24,0% (n=44) que a criança tinha um horário por dia para usar o dispositivo (Tabela 1), enquanto 91,8% (n=168) concordaram que o pai estava presente durante o uso do dispositivo de tela (Tabela 3). A mediana geral do tempo de uso foi significativamente maior para as crianças que usaram o dispositivo móvel sem a presença do pai em comparação com aquelas que o usaram com o pai presente (p-valor = 0,042) (Tabela 4). Em termos de percepções de uso e frequência de uso, 48,1% (n=88) dos entrevistados concordaram que a criança usava o dispositivo móvel por mais tempo do que deveria e 17,5% (n=32) concordaram que a criança achava mais divertido usar o *smartphone* ou *tablet* do que fazer outras atividades (por exemplo, praticar esportes, ler, brincar no recreio) (Tabela 1). Cerca de 17,5% (n=32) e 2,7% (n=5) dos entrevistados usaram o dispositivo móvel para manter a criança entretida em diferentes ambientes e como recompensa ou punição. (Tabela 3). A mediana geral do tempo de uso foi significativamente maior nos filhos de pais que usaram essas estratégias em comparação com os que não usaram (p-valor <0,001 e p-valor 0,008, respectivamente) (Tabela 4). 

## Discussão

 De acordo com nossos resultados, os pais e responsáveis pelas crianças de nossa amostra parecem estar preocupados, em geral, com o uso e o tempo de tela gasto pelas crianças em dispositivos móveis ( *smartphone* e/ou *tablet* ). O tempo de tela durante a primeira infância tem sido uma preocupação pediátrica e de saúde pública ^(^ 2023 ^)^ . Esse pode ser um dos fatores que contribuem para as disparidades no desenvolvimento da primeira infância. 

 A norma parental de acesso irrestrito das crianças a dispositivos móveis está associada ao aumento do tempo de uso diário, sendo 2,6 vezes maior em comparação com aqueles sem acesso livre, o que pode ter consequências para o desenvolvimento cognitivo, social e emocional das crianças ^(^ 2023 ^)^ . O fato de as crianças com menos de dois anos de idade usarem dispositivos móveis mais de 30 minutos por dia, e de esse tempo aumentar consideravelmente quando elas têm acesso livre, não atende a nenhuma recomendação de tempo de tela e levanta preocupações sobre o impacto da exposição precoce à tecnologia no desenvolvimento infantil ^(^ 2023 ^)^ . Uma proporção muito baixa de pais usa o *smartphone* ou *tablet* como recompensa ou punição, com uso médio de duas horas por dia, dependendo do comportamento da criança. É reconfortante o fato de que a maioria não recorre a essa estratégia disciplinar, mas aqueles que a utilizam para não cumprir as recomendações são preocupantes. 

**Tabela 1:** - Contagens e porcentagens gerais e estratificadas da concordância dos pais e responsáveis com as normas de uso de dispositivos móveis ( *smartphone* / *tablet* ) e percepções da frequência de uso e do comportamento da criança durante ou após o uso (n = 183). Espanha, 2021-2022

	**n**	Q1* n (%)	**p** ^†^	**(95% IC)** ^‡^	**Q2** ^§^ **n (%)**	**p** ^†^	**(95% IC)** ^‡^	**Q3** ^||^ **n (%)**	**p** ^†^	**(95% IC)** ^‡^	**Q4** ^¶^ **n (%)**	**p** ^†^	**(95% IC)** ^‡^
Total	183	3 (1,6)			7 (3,8)			34 (18,6)			44 (24,0)		
Sexo da criança			0,305	(-0,08 a 0,01)		0,581	(-0,09 a 0,04)		0,421	(-0,08 a 0,01)		0,103	(-0,24 a 0,01)
Masculino	99	3 (3,0)			5 (5,1)			21 (21,2)			29 (29,3)		
Feminino	84	0 (0,0)			2 (2,4)			13 (15,5)			15 (17,9)		
Idade da criança			1,000	(-0,06 a 0,04)		0,801	(-0,04 a 0,09)		0,006	(0,05 a 0,28)		0,874	(-0,15 a 0,11)
Menor ou igual a 2 anos de idade	100	2 (2,0)			3 (3,0)			11 (11,0)			25 (25,0)		
De 3 a 5 anos de idade	83	1 (1,2)			4 (4,8)			23 (27,7)			19 (22,9)		
Irmãos			0,946	(-0,06 a 0,03)		0,099	(-0,13 a -0,0018		0,050	(-0,24 a -0,01)		0,819	(-0,10 a 0,15)
Sim	88	2 (2,3)			6 (6,9)			22 (25,0)			20 (22,7)		
Não	95	1 (1,1)			1 (1,1)			12 (12,6)			24 (25,3)		
Irmãos mais velhos			0,567	(-0,09 a 0,02)		0,089	(-0,15 a 0,00)		0,055	(-0,26 -0,01)		0,813	(-0,11 a 0,15)
Sim	63	2 (3,2)			5 (7,9)			17 (26,9)			14 (22,2)		
Não	120	1 (0,8)			2 (1,7)			17 (14,2)			30 (25,0)		
Relacionamento com a criança			1,000	(-0,05 a 0,11)		0,831	(-0,08 a 0,09)		0,727	(-0,14 a 0,24)		1,000	(-0,16 a 0,24)
Mãe	163	3 (1,8)			7 (4,3)			30 (18,4)			39 (23,9)		
Pai	17	0 (0,0)	-		0 (0,0)			2 (11,8)	-		4 (23,5)	-	
Outro	3	0 (0,0)			0 (0,0)			2 (66,7)			1 (33,3)		
Idade do entrevistado			0,225	(-0,09 a 0,01)		0,398	(-0,10 a 0,03)		0,124	(-0,01 a 0,21)		0,315	(-0,05 a 0,20)
Menor ou igual a 35 anos de idade	89	3 (3,4)			5 (5,6)			12 (13,5)			18 (20,2)		
Mais de 35 anos de idade	94	0 (0,0)			2 (2,1)			22 (23,4)			26 (27,7)		
Nível de escolaridade			0,234	(-0,15 a 0,09)		1,000	(-0,12 a 0,05)		0,158	-0,28 a 0,03)		0,639	(-0,11 a 0,18)
Inferior, primário ou secundário	40	2 (5,0)			2 (5,0)			11 (27,5)			8 (20,0)		
Estudos universitários	143	1 (0,7)			5 (3,5)			23 (16,1)			36 (25,2)		
	**n**	Q5** n (%)	**p** ^†^	**(95% IC)** ^‡^	**Q6** ^††^ **n (%)**	**p** ^†^	**(95% IC)** ^‡^	**Q7** ^‡‡^ **n (%)**	**p** ^†^	**(95% IC)** ^‡^	**Q8** ^§§^ **n (%)**	**p** ^†^	**(95% IC)** ^‡^
Total	183	32 (17,5)			88 (48,1)			100 (54,6)			54 (29,5)		
Sexo da criança			0,941	(-0,13 a 0,10)		0,080	(-0,28 a 0,01)		1,000	(-0,14 a 0,15)		0,456	(-0,19 a 0,07)
Masculino	99	18 (18,2)			54 (54,6)			54 (54,6)			32 (32,3)		
Feminino	84	14 (16,7)			34 (40,5)			46 (54,8)			22 (26,2)		
Idade da criança			0,431	(-0,17 a 0,06)		0,286	(-0,06 a 0,23)		0,124	(-0,02 a 0,27)		0,051	(-0,27 a -0,01)
Menor ou igual a 2 anos de idade	100	20 (20,0)			44 (44,0)			49 (49,00)			36 (36,0)		
De 3 a 5 anos de idade	83	12 (14,5)			44 (53,0)			51 (61,5)			18 (21,7)		
Irmãos			0,410	(-0,17 a 0,05)		0,067	(-0,29 a -0,00)		0,056	(-0,29 a -0,01)		0,862	(-0,16 a 0,11)
Sim	88	18 (20,5)			49 (55,7)			55 (62,5)			27 (30,7)		
Não	95	14 (14,7)			39 (41,1)			45 (47,4)			27 (28,4)		
Irmãos mais velhos			0,543	(-0,18 a 0,07)		0,053	(-0,31 -0,01)		0,202	(-0,26 a 0,04)		1,000	(-0,15 a 0,13)
Sim	63	13 (20,6)			37 (58,7)			39 (61,9)			19 (30,2)		
Não	120	19 (15,8)			51 (42,5)			61 (50,8)			35 (29,2)		
Relacionamento com a criança			0,288	(-0,04 a 0,37)		0,381	(-0,35 a 0,09)		0,014	(-0,46 a -0,04)		0,823	(-0,18 a 0,24)
Mãe	163	26 (15,9)			81 (49,7)			94 (57,7)			48 (29,5)		
Pai	17	5 (29,4)	-		6 (35,3)	-		4 (23,5)	-		6 (35,3)	-	
Outro	3	1 (33,3)			1 (33,3)			2 (66,7)			0 (0,0)		
Idade do entrevistado			0,678	(-0,08 a 0,15)		1,000	(-0,15 a 0,14)		0,579	(-0,20 a 0,09)		0,567	(-0,08 a 0,18)
Menor ou igual a 35 anos de idade	89	14 (15,7)			43 (48,3)			51 (57,3)			24 (26,9)		
Mais de 35 anos de idade	94	18 (19,2)			45 (47,9)			49 (52,1)			30 (31,9)		
Nível de escolaridade			0,811	(-0,19 a 0,09)		0,924	(-0,20 a 0,15)		0,625	(-0,11 a 0,23)		0,366	(-0,07 a 0,23)
Inferior, primário ou secundário	40	8 (20,0)			20 (50,0)			20 (50,0)			9 (22,5)		
Estudos universitários	143	24 (17,8)			68 (47,6)			80 (55,9)			45 (31,5)		

* Q1 = Seu filho pode usar o *smartphone* ou *tablet* sem limite de tempo

^†^ p = p- *value* (Cada valor de p vem do valor do qui-quadrado pelo teste de Fisher)

^‡^ 95%IC = 95% Intervalo de confiança

^§^ Q2 = Seu filho pode usar o *smartphone* ou *tablet* sem limitação de tempo nos finais de semana

^||^ Q3 = Seu filho pode usar o *smartphone* ou *tablet* somente nos finais de semana

^¶^ Q4 = Seu filho tem um horário por dia para usar o *smartphone* ou *tablet*

** Q5 = Seu filho acha mais divertido usar o *smartphone* ou *tablet* do que fazer outras atividades, como esportes, leitura, brincar no recreio ou outras atividades

^††^ Q6 = Seu filho usa o *smartphone* ou *tablet* por mais tempo do que deveria

^‡‡^ Q7 = Seu filho fica quieto quando para de usar o *smartphone* ou *tablet*

^§§^ Q8 = Seu filho chora ou grita quando deixa de usar o *smartphone* ou *tablet*

Tabela 2 - Medianas e quartis gerais e estratificados por dispositivo do tempo de uso (em minutos por dia) em crianças, de acordo com a concordância com as normas de uso de dispositivos móveis e com as percepções da frequência de uso e do comportamento da criança durante ou após o uso, de acordo com os pais e responsáveis (n = 183). Espanha, 2021-2022

**Table:** 

	***Smartphone* e/ou *tablet* (n=183)**	**Somente *smartphone* (n=139)**	**Somente *tablet* (n=80)**
	Mediana (IQR*)	Mediana (IQR*)	Mediana (IQR*)
Q1 ^†^			
Concordo ^‡^	103,0 (90,0-111,0)	77,1 (68,6-90,0)	60,0 (60,0-60,0)
Rest ^§^	30,0 (17,1-51,4)	30,0 (8,57-30,0)	30,0 (17,1-60,0)
p ^||^	0,010	0,005	0,912
Q2 ^¶^			
Concordo ^‡^	55,7 (27,9-90,0)	60,0 (30,0-77,1)	17,1 (8,57-47,1)
Rest ^§^	30,0 (17,1-51,4)	30,0 (8,57-30,0)	30,0 (17,1-62,1)
p ^||^	0,164	0,402	0,305
Q3**			
Concordo ^‡^	23,6 (8,6-54,6)	17,1 (8,57-34,3)	21,4 (8,6-55,7)
Rest ^§^	30,0 (21,4-55,7)	30,0 (17,1-38,6)	30,0 (24,6-65,4)
p ^||^	0,102	0,026	0,167
Q4 ^††^			
Concordo ^‡^	30,0 (8,6-55,7)	27,9 (8,6-30,0)	30,0 (23,6-60,0)
Rest ^§^	30,0 (19,3-53,6)	30,0 (17,1-38,6)	30,0 (17,1-60,0)
p ^||^	0,527	0,012	0,215
Q5 ^‡‡^			
Concordo ^‡^	34,3 (17,1-63,2)	30,0 (8,57-38,6)	34,3 (25,7-58,9)
Rest ^§^	30,0 (17,1-40,7)	30,0 (8,57-30,0)	30,0 (10,7-60,0)
p ^||^	0,256	0,773	0,101
Q6 ^§§^			
Concordo ^‡^	38,6 (30,0-66,4)	30,0 (21,43-60,0)	34,3 (17,1-73,9)
Rest ^§^	30,0 (8,6-30,0)	30,0 (8,57-30,0)	30,0 (8,57-40,7)
p	< 0,001	0,005	0,038
Q7 ^||||^			
Concordo ^‡^	30,0 (20,4-60,0)	30,0 (8,57-38,6)	30,0 (22,5-71,8)
Rest ^§^	30,0 (17,1-38,6)	30,0 (8,57-30,0)	30,0 (8,57-50,4)
p ^||^	0,047	0,627	0,024
Q8 ^¶¶^			
Concordo ^‡^	30,0 (17,1-48,2)	30,0 (17,1-38,6)	30,0 (8,57-60,0)
Rest ^§^	30,0 (17,1-55,7)	30,0 (8,57-30,0)	30,0 (24,6-61,1)
p ^||^	0,970	0,702	0,277

* IQR = Intervalo interquartil

^†^ Q1 = Seu filho pode usar o *smartphone* ou *tablet* sem limite de tempo

^‡^ Concordo = Concordo plenamente + Concordo

^§^ Rest = Não concordo nem discordo + Discordo + Discordo totalmente

^||^ p = p- *value* (Cada valor de p é do teste U de Mann-Whitney)

^¶^ Q2 = Seu filho pode usar o *smartphone* ou *tablet* sem limitação de tempo nos finais de semana

** Q3 = Seu filho pode usar o *smartphone* ou *tablet* somente nos finais de semana

^††^ Q4 = Seu filho tem um horário por dia para usar o *smartphone* ou *tablet*

^‡‡^ Q5 = Seu filho acha mais divertido usar o *smartphone* ou *tablet* do que fazer outras atividades, como esportes, leitura, brincar no recreio ou outras atividades

^§§^ Q6 = Seu filho usa o *smartphone* ou *tablet* por mais tempo do que deveria

^||||^ Q7 = Seu filho fica quieto quando deixa de usar o *smartphone* ou *tablet*

^¶¶^ Q8 = Seu filho chora ou grita quando deixa de usar o *smartphone* ou *tablet*

 A presença dos pais no uso de dispositivos foi associada a ter um irmão, mas não a ter um irmão mais velho. Outro estudo indicou que a presença de um irmão e menos espaço pessoal na sala de estar foram considerados preditores significativos do tempo de tela das crianças ^(^ 2021 ^)^ . O gerenciamento do uso de *smartphone* s orientado pelo controle dos pais não é muito eficaz e agrava o vício em *smartphone* s, considerando que os pais que passam mais tempo na frente da tela percebem o tempo de tela dos filhos com menos preocupação ^(^ 2019 ^,^ 2021 ^)^ . Isso sugere que os fatores socioculturais e ambientais do lar e da comunidade, além dos fatores comportamentais dos pais, 

 Tabela 3 - Contagens e porcentagens de concordância com estratégias de uso de dispositivos móveis ( *smartphone* / *tablet* ) para manter as crianças entretidas em diferentes ambientes, uso de dispositivos móveis como recompensa ou punição, em pais e responsáveis (n = 183). Espanha, 2021-2022 

**Table:** 

	**n**	Uso do dispositivo móvel como entretenimento em diferentes ambientes * n (%)	**p** ^†^	**(95% IC)** ^‡^	**Uso do dispositivo móvel como recompensa/punição n (%)**	**p** ^†^	**(95% IC)** ^‡^	**Presença dos pais durante o uso do dispositivo móvel n (%)**	**p** ^†^	**(95% IC)** ^‡^
Total	183	32 (17,5)			5 (2,7)			168 (91,8)		
Sexo da criança			0,213	(-0,19 a 0,03)		1,000	(-0,06 a 0,05)		0,453	(-0,04 a 0,12)
Masculino	99	21 (21,2)			3 (3,0)			89 (89,0)		
Feminino	84	11 (13,1)			2 (2,4)			79 (94,0)		
Idade da criança			0,437	(-0,06 a 0,17)		0,832	(-0,04 a 0,08)		0,144	(-0,16 a 0,01)
Menor ou igual a 2 anos de idade	100	15 (15,0)			2 (2,0)			95 (95,0)		
De 3 a 5 anos de idade	83	17 (20,5)			3 (3,6)			73 (88,0)		
Irmãos			0,410	(-0,17 a 0,05)		0,320	(-0,10 a 0,02)		0,020	(0,03 a 0,19)
Sim	88	18 (20,5)			4 (4,5)			76 (86,4)		
Não	95	14 (14,7)			1 (1,1)			92 (96,8)		
Irmãos mais velhos			0,153	(-0,23 a 0,02)		0,089	(-0,14 a -0,00)		1,000	(-0,09 a 0,09)
Sim	63	15 (23,8)			4 (6,3)			58 (92,1)		
Não	120	17 (14,2)			1 (0,8)			110 (91,7)		
Relacionamento com a criança			0,750	(-0,12 a 0,25)		1,000	(-0,07 a 0,10)		0,004	(-0,41 a -0,03)
Mãe	163	28 (17,2)			5 (3,1)			153 (93,9)		
Pai	17	4 (23,5)			0 (0,0)			12 (70,6)		
Outro	3	0 (0,0)			0 (0,0)			3 (100,0)		
Idade do entrevistado			0,006	(-0,27 a -0,05)		0,060	(-0,12 a -0,01)		1,000	(-0,09 a 0,08)
Menor ou igual a 35 anos de idade	89	23 (25,8)			5 (5,6)			82 (92,1)		
Mais de 35 anos de idade	94	9 (9,6)			0 (0,0)			86 (91,5)		
Nível de escolaridade			0,238	(-0,26 a 0,04)		1,000	(-0,09 a 0,05)		0,885	(-0,07 a 0,15)
Inferior, primário e secundário	40	10 (25,0)			1 (2,5)			36 (90,0)		
Universidade	143	22 (15,4)			4 (2,8)			132 (92,3)		

* Diferentes Situações = Restaurantes, consultas médicas, transporte público ou outros

^†^ p = p- *value* (cada valor de p vem do valor do qui-quadrado pelo teste de Fisher)

^‡^ 95%IC = 95% Intervalo de confiança

Tabela 4 - Medianas e quartis de tempo (em minutos por dia) de uso de acordo com o dispositivo móvel em crianças e estratégias diárias de uso de dispositivos móveis, uso como recompensa/punição e presença dos pais, em pais e responsáveis (n = 183). Espanha, 2021-2022

**Table:** 

	***Smartphone* e *tablet***	**Somente *smartphone***	**Somente *tablet***
	Mediana (IQR*)	Mediana (IQR*)	Mediana (IQR*)
Uso do dispositivo móvel como entretenimento em diferentes situações ^†^			
Concordo ^‡^	49,3 (30,0-80,4)	30,0 (17,1-47,1)	53,6 (28,9-90,0)
Rest ^§^	30,0 (17,1-38,6)	30,0 (8,57-30,0)	30,0 (10,7-55,7)
p ^||^	< 0,001	< 0,001	0,510
Uso do dispositivo móvel como recompensa/punição			
Concordo ^‡^	120,0 (55,7-163,0)	30,0 (30,0-60,0)	81,4 (51,4-114,0)
Rest ^§^	30,0 (17,1-50,4)	30 (8,57-36,4)	30,0 (15,0-60,0)
p ^||^	0,008	0,051	0,020
Presença dos pais durante o uso do dispositivo móvel			
Concordo ^‡^	30,0 (17,1-48,2)	30,0 (8,57-30,0)	30,0 (8,57-60,0)
Rest ^§^	47,1 (23,6-101,0)	30,0 (8,57-60,0)	38,6 (25,7-75,0)
p ^||^	0,042	0,668	0,005

* IQR = Intervalo interquartil

^†^ Diferentes Situações = Restaurantes, consultas médicas, transporte público ou outros

^‡^ Concordo = Concordo plenamente +Concordo

^§^ Rest = Não concordo nem discordo + Discordo + Discordo totalmente

^||^ p = p- *value* (cada valor de p é do teste U de Mann-Whitney)

 como o conhecimento sobre os efeitos dos dispositivos móveis na saúde, podem favorecer o uso pelas crianças. As instituições de saúde e a equipe de saúde, especialmente os enfermeiros, devem assumir o desafio de conscientizar as crianças e os adolescentes sobre o uso responsável de dispositivos móveis ^(^ 2021 ^)^ . Ainda mais se considerarmos que, à medida que as crianças crescem, o nível de autonomia e independência no uso de dispositivos móveis também aumenta, o que coincide com os resultados deste estudo ^(^ 2017 ^,^ 2024 ^)^ . 

 Os pais mais velhos, que relatam mais tempo de uso de dispositivos móveis por seus filhos, podem enfrentar desafios adicionais em termos de estabelecimento de limites e promoção do equilíbrio entre o tempo de tela e outras atividades, pois foi evidenciado que quanto maior a idade dos pais e quanto mais tempo os pais passam vendo dispositivos móveis, maior o tempo de tela das crianças ^(^ 2023 ^)^ . As diferenças no tempo gasto com dispositivos móveis podem refletir diferentes atitudes e percepções em relação à tecnologia nos pais mais velhos, que podem ter uma visão mais permissiva ou valorizar mais os possíveis benefícios da tecnologia. Por outro lado, os pais mais jovens podem ser mais cautelosos ou restritivos em relação ao tempo gasto com dispositivos móveis. Favoravelmente, a maioria dos pais (91,8%) está presente enquanto seus filhos estão usando dispositivos móveis, indicando supervisão ativa e permissividade dos pais durante o uso de dispositivos móveis. Encontramos associações, como o uso de dispositivos móveis exclusivamente nos fins de semana, com a idade da criança e o relacionamento com ela (mãe ou pai), o que se explica pelo fato de a sociedade espanhola ter passado por mudanças drásticas no estilo de vida nas últimas décadas. 

 Outra associação foi a percepção de que a criança permanece calma quando para de usar o dispositivo móvel e o relacionamento com a criança (mãe ou pai), o que aponta para a ideia de que alguns pais acreditam que o uso pode ter um impacto positivo no comportamento de seus filhos. As associações mostram que esse novo determinante da saúde exige intervenções diferentes, tendo em vista os riscos envolvidos. Quase um quarto das crianças espanholas com idade entre um e 14 anos que excederam 120 minutos de tempo diário de tela em atividades de lazer tiveram uma redução na duração do sono ^(^ 2021a ^)^ . 

 Até o momento, os estudos não se concentraram em estudar as normas e as percepções dos pais e responsáveis na Europa em relação ao uso de dispositivos de tela. Quase todos são encontrados em países asiáticos e anglo-saxões ^(^ 2023 ^,^ 2021 ^,^ 2023 ^,^ 2011 ^)^ . Até onde sabemos, este é um dos primeiros estudos que descreve as normas e percepções dos pais de crianças que usam dispositivos móveis, deixando para trás o paradigma da televisão. A inclusão de pais, mães e responsáveis, bem como a variabilidade nos níveis educacionais, oferece uma perspectiva ampla e permite resultados mais gerais. A combinação de dados numéricos e respostas perceptivas proporciona uma compreensão mais completa do uso de dispositivos móveis pelas crianças. O estudo aborda a questão do acesso e do uso de dispositivos móveis pelas crianças, que é relevante atualmente e pode ter implicações para a criação dos filhos e o desenvolvimento infantil. 

 No futuro, espera-se um aumento drástico no uso de dispositivos móveis pelas crianças. Isso ressalta a importância de fornecer educação e apoio aos pais em relação ao uso da tela durante os primeiros anos de vida, considerando as realidades da sociedade atual ^(^ 2023 ^)^ . Há evidências de que os enfermeiros podem intervir no âmbito da educação dos pais para o cumprimento das recomendações sobre o tempo de tela em crianças, levando em conta que esse é um determinante social da saúde em nossos tempos ^(^ 2023 ^,^ 2022 ^)^ . A colaboração entre pesquisadores, formuladores de políticas e pais é essencial para atualizar as recomendações para pediatras e enfermeiros para gerenciar adequadamente o tempo de tela durante a primeira infância ^(^ 2021 ^,^ 2023 ^,^ 2024 ^)^ . 

 O presente estudo tem algumas limitações. Em primeiro lugar, a seleção dos participantes pode ter gerado vieses e limitado a representatividade da amostra. Além disso, não perguntamos sobre o uso de dispositivos móveis pelos pais, o que poderia influenciar o tempo de uso dos filhos ^(^ 2023 ^,^ 2019 ^)^ . Não são fornecidas informações sobre o processo de desenvolvimento e validação do questionário, o que levanta dúvidas sobre a confiabilidade e a validade das medidas usadas. A avaliação do tempo de uso em intervalos de 30 minutos pode ser imprecisa e depender da memória dos participantes. Na última categoria, limitamos o tempo de uso ao máximo de quatro horas. Portanto, se houver crianças que tenham usado mais do que esse tempo, não as levamos em consideração. Este estudo não verificou os aspectos qualitativos do fenômeno e foi um autorrelato do participante sobre o tempo de uso de dispositivos móveis em crianças, pois considerou a declaração do cuidador; no entanto, isso apoia a ideia de mais pesquisas sobre esse aspecto. 

## Conclusão

As regras dos pais influenciam o tempo que as crianças passam em dispositivos móveis. Permissões ilimitadas resultam em mais tempo de tela, com possíveis efeitos sobre o desenvolvimento das crianças. A supervisão dos pais também é importante; sem ela, as crianças passam mais tempo em frente às telas. Muitos pais admitem que seus filhos excedem os limites recomendados e preferem os dispositivos a outras atividades. Essas percepções influenciam o tempo de tela, destacando a necessidade de supervisão dos pais e educação precoce. Os profissionais de saúde, como os enfermeiros, podem desempenhar um papel fundamental em duas áreas principais: educar os pais para que cumpram as recomendações e promover padrões saudáveis de tempo de tela em crianças e adolescentes.
